# Poly(aniline-*co*-melamine)@MnFe_2_O_4_ nanocatalyst for the synthesis of 4,4′-(arylmethylene) bis (1H-pyrazole-5-ol) derivatives, and 1,4- dihydropyrano[2,3-*c*]pyrazoles and evaluation of their antioxidant, and anticancer activities

**DOI:** 10.3389/fchem.2022.1046120

**Published:** 2022-10-28

**Authors:** Shefa Mirani Nezhad, Seied Ali Pourmousavi, Ehsan Nazarzadeh Zare, Golnaz Heidari, Hamed Manoochehri, Esmaeel Sharifi

**Affiliations:** ^1^ School of Chemistry, Damghan University, Damghan, Iran; ^2^ Research Center for Molecular Medicine, Hamadan University of Medical Sciences, Hamadan, Iran; ^3^ Department of Tissue Engineering and Biomaterials, School of Advanced Medical Sciences and Technologies, Hamadan University of Medical Sciences, Hamadan, Iran

**Keywords:** poly(aniline-co-melamine)@MnFe2O4 nanocomposite, antioxidant, anticancer, pyrazole derivatives, nanocatalyst

## Abstract

In this work, magnetic poly(aniline-*co*-melamine) nanocomposite as an efficient heterogeneous polymer-based nanocatalyst was fabricated in two steps. First, poly(aniline-*co*-melamine) was synthesized through the chemical oxidation by ammonium persulfate, then the magnetic nanocatalyst was successfully prepared from the *in-situ* coprecipitation method in the presence of poly(aniline-*co*-melamine). The resulting poly(aniline-*co*-melamine)@MnFe_2_O_4_ was characterized by FTIR, FESEM, XRD, VSM, EDX, TGA, and UV-vis analyses. The catalytic activity of poly(aniline-*co*-melamine)@MnFe_2_O_4_ was investigated in the synthesis of 4,4′-(arylmethylene)bis(1H-pyrazole-5-ol) derivatives, and new alkylene bridging bis 4,4′-(arylmethylene)bis(1H-pyrazole-5-ol) derivatives in excellent yields. The yield of 1,4-dihydropyrano[2,3-c]pyrazoles, 4,4′-(arylmethylene)bis(1H-pyrazol-5-ol), yields, and new alkylene bridging bis 4,4′-(arylmethylene)bis(1H-pyrazol-5-ol) derivatives were obtained 89%–96%, 90%–96%, and 92%–96%, respectively. The poly(aniline-*co*-melamine)@MnFe_2_O_4_ nanocatalyst can be recycled without pre-activation and reloaded up to five consecutive runs without a significant decrease in its efficiency. In addition, the antioxidant activity of some derivatives was evaluated by DPPH assay. Results showed that the maximum antioxidant activity of 4,4′-(arylmethylene)bis(1H-pyrazole-5-ol) derivatives and 1,4-dihydropyrano[2,3-*c*]pyrazoles were 75% and 90%, respectively. Furthermore, 4,4′-(arylmethylene)bis(1H-pyrazole-5-ol) derivatives and 1,4-dihydropyrano[2,3-*c*]pyrazoles showed good potential for destroying colon cancer cell lines. Consequently, the poly(aniline-*co*-melamine)@MnFe_2_O_4_ nanocomposite is an excellent catalyst for green chemical processes owing to its high catalytic activity, stability, and reusability.

## 1 Introduction

Magnetic nanoparticles have been widely used as viable catalyst supports due to their high surface area, which results in high catalyst loading capacity, great dispersion, remarkable stability, and easy catalytic recycling (Zhu et al., 2010; Guo et al., 2021a; Lian et al., 2021; Soares et al., 2022). The recovery of catalysts from liquid-phase processes is significantly easier with magnetic separation than with cross-flow filtering and centrifugation (Gu et al., 2020; Dang et al., 2022; Xu et al., 2022). Additionally, except for those that are very corrosive or acidic, the particles’ magnetic characteristics are persistent enough to withstand the majority of chemical conditions. Easy accessibility of reactants to the active sites is also possible since the catalysts are often bound to the surface of the magnetic nanoparticles (Stevens et al., 2005; Hassanzadeh-Afruzi et al., 2022; Movagharnezhad et al., 2022). Because of their electrical resistance, physical and magnetic characteristics, and great chemical stability, ferrites, a subclass of ferrimagnetic ceramics, are widely recognized (Alizadeh and Baseri, 2022). The spinel, hexagonal, and garnet structures of ferrites are determined by their original crystal lattice. The normal and inverse spinel ferrites are particularly appealing among these structures (Bououdina and Manoharan, 2020; Özdemir et al., 2020).

Superparamagnetism, single domain effects, high coercivity, moderate magnetization, and spin-filtering are just a few of the distinctive magnetic properties that make it possible for magnetic transition oxide nanostructures with spinel structure MFe_2_O_4_ (M = Ni, Co, Fe, Mn, etc.) to have remarkable biological and industrial functionalities (Zipare et al., 2015). Due to its captivating electromagnetic and magnetic characteristics, as a significant member of the ferrite family, MnFe_2_O_4_ (MFO) has garnered significant study attention. As MnFe_2_O_4_ is partly inverse spinel, about 80% of Mn^2+^ ions are found at the tetrahedral site, while just 20% are found at the octahedral site (Deraz and Alarifi, 2012). The features of MnFe_2_O_4_ nanoparticles and thin films are varied, including high curie temperature, superparamagnetism, super spin glass state, size-dependent saturation magnetization, and high anisotropy constant (Carta et al., 2010). The magnetic nanoparticles such as MnFe_2_O_4_, MgFe_2_O_4_, CoFe_2_O_4_, ZnFe_2_O_4_, Fe_3_O_4_, and their nanocomposites due to their biocompatibility, good physicochemical properties and small particle size have several applications in targeted drug delivery, magnetic resonance imaging (MRI), mediators for hyperthermia applications, etc., (Islam et al., 2020).

The magnetic particles are rarely used in their unprocessed form; instead, they are often mixed with an organic polymer matrix or an inorganic silica carrier and placed in a liquid media to prevent aggregation (Guo et al., 2021b; Jazzar et al., 2021). The particles are endowed with organic polymers with the functional groups required for the desired uses (Kalia et al., 2014). This combination of organic polymers and magnetic nanoparticles provides a diverse range of opportunities for the creation of a new class of organic/inorganic hybrid materials that have dual properties. The resulting magnetic polymers have shown to be helpful for a range of purposes since they combine magnetic characteristics with high stability and reputable biocompatibility. The polymer nanocomposites due to high functionality, good phsicochmichal proprtes, and biocompatibility have received increasing attention in biomedical applications (Hule and Pochan, 2007). Thus, they’ve been used effectively in new applications including hyperthermia, bioseparation, medication delivery, targeted oil recovery, and so on (Philippova et al., 2011). Nevertheless, since magnetic nanoparticles possess a high surface-area-to-volume ratio and are scattered in a polymer matrix, it is difficult to create magnetic polymer nanocomposites that have a consistent magnetic field equivalent to that of an inorganic magnetic material. It is commonly known that a particle’s magnetic properties depend on its chemical composition, shape, size, and surface characteristics. To determine the sorts of applications appropriate for polymer usage, precise control of the aforementioned factors is required. The creation of magnetic polymer nanocomposites has been investigated using a variety of methods, including simultaneous formation of polymers and particles, polymerization of monomers with the presence of particles, *in-situ* formation of particles into preformed polymers, and direct mixing (Massart, 1981; Jazzar et al., 2021).

Pyrazoles, commonly known as azoles, are five-membered ring heterocyclic compounds that include two nitrogen atoms in neighboring locations (Parajuli et al., 2013). Treatment of inflammation and illnesses linked to inflammation, such as arthritis, is the most well-known use of nearly every category of pyrazoles. Pyrazoles are the focus of numerous investigations due to their broad range of biological potential activities, including anticancer, anti-inflammatory (Amir et al., 2008), antihistaminic (Yildirim et al., 2005), antitumor (Park et al., 2005), antiviral (Larsen et al., 1999), anti-diabetic, anticonvulsant, and antimicrobial (Pimenova and Voronina, 2001) activities. Ethylacetoacetate and substituted hydrazine are typically used as the starting materials when synthesizing pyrazolone. With the aid of numerous catalysts, the production of pyrazoles has been reported to employ a variety of techniques and conditions. For example, pyrazolo[3,4-b]pyridine-5-carboxamide derivatives were catalyzed by p-toluenesulfonic acid under mild reaction conditions and in good yields at ambient temperature (Shaabani et al., 2009), Poly(ethylene imine)-modified magnetic halloysite nanotubes was used as a catalyst for the synthesis of dihydropyrano[2,3-c] pyrazole derivatives (Hajizadeh and Maleki, 2018).

In this study, we focused on fabricating magnetic poly(aniline-*co*-melamine)@MnFe_2_O_4_ nanocomposite as a catalyst for the syntheses of 1,4-dihydropyrano[2,3-*c*]pyrazoles and synthesis of 4,4′-(arylmethylene)bis(1H-pyrazole-5-ols). These compounds were also tested for antioxidant and anticancer activities.

## 2 Experimental section

### 2.1 Materials and methods

Aniline, melamine, ferric chloride (FeCl_3_·6H_2_O), ammonium persulfate, manganese (II) chloride (MnCl_2_.2H_2_O), sodium hydroxide, solvents, and all other reagents were purchased from Merck Company, Germany.

### 2.2 Preparation of poly(aniline-*co*-melamine)@MnFe_2_O_4_ nanocatalyst

**FIGURE 1:**
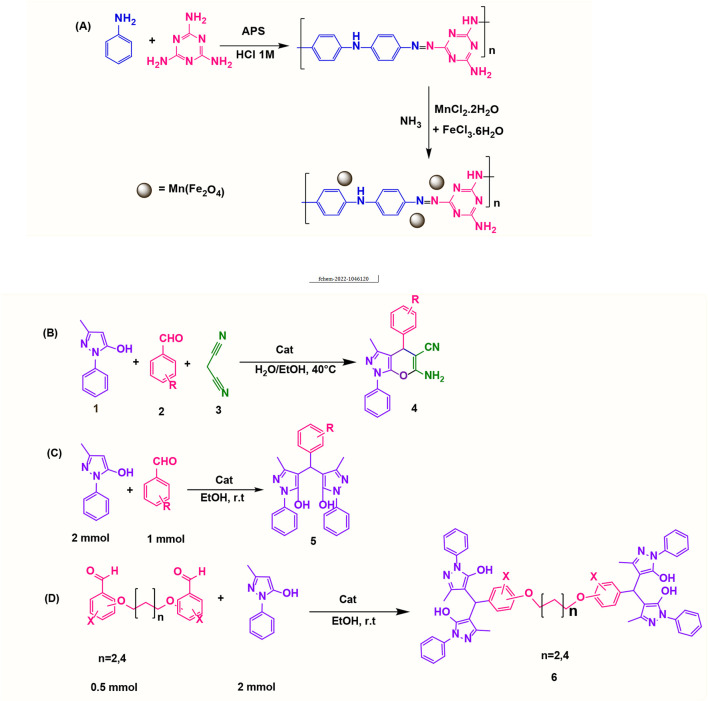
Preparation of poly(aniline-*co*-melamine)@MnFe_2_O_4_ nanocomposite in two steps **(A)**, synthesis of 1,4-dihydropyrano[2,3-*c*]pyrazoles **(B)**, synthesis of 4,4′-(arylmethylene)bis(1H-pyrazole-5-ol) derivatives **(C)**, and synthesis of bis 4,4′-(arylmethylene)bis(1H-pyrazole-5-ol) derivatives **(D)**.

To 40 ml of HCl (1 M), 1 ml aniline and 1.38 g melamine were added. The reaction mixture was stirred at 50°C until the melamine was entirely dissolved and was kept at 0°C–4°C under an inerth atmosphere. Then, 20 ml of ammonium persulfate solution (6.02 g) was added dropwise to the above solution. The reaction was maintained at 0°C–4°C for 12 h. The dark green poly(aniline-*co*-melamine) precipitate was then recovered by filtering and washed multiple times using water and methanol to eliminate the residual oxidizing agent and oligomers. The cleansed product was submerged in boiling water for 20 min and agitated to thoroughly dissolve the undissolved melamine. The *in-situ* co-precipitation method was used for the preparation of poly(aniline-*co*-melamine)@MnFe_2_O_4_ nanocomposite as follows:

0.33 g of MnCl_2_.2H_2_O and 0.5 g of FeCl_3_.6H_2_O were dissolved in 25 ml of deionized water. The solution was then mixed with 1 g of poly(aniline-*co*-melamine) at 80°C for 20 min. The mixture was treated with a 1 M NaOH solution until the pH reached 12. After 3 h, the black precipitate was magnetically separated and repeatedly rinsed with deionized water and ethanol and drided at 40°C in the vacuum oven for 10 h. Figure 1A shows the reaction steps for the preparation of the nanocomposite.

### 2.3 General procedure for the synthesis of 3-methyl-1-phenyl-1H-pyrazole-5-ol

For the synthesis of 3-methyl-1-phenyl-1H-pyrazole-5-ol, phenylhydrazine (0.05 mol, 5 ml), acetic acid (0.5 ml), and ethyl acetoacetate (0.05 mol, 6.2 ml) were stirred in the reaction vessel at 90°C for 1 hour. After cooling at room temperature, 10–20 ml ether was added to the mixture the precipitate was filtered, and the product was crystallized using ethanol (Nezhad et al., 2022).

### 2.4 General procedure for the synthesis of 1,4-dihydropyrano[2,3-*c*]pyrazole derivatives

A mixture of 3-methyl-1-phenyl-1*H*-pyrazole-5-ol (1 mmol), an aldehyde (1.0 mmol), malononitrile (1 mmol), and poly(aniline-*co*-melamine)@MnFe_2_O_4_ nanocomposite (0.05 g) in water/EtOH (5 ml 1:1) was stirred at 40°C until the reaction was complete. The completion of the reaction was monitored by thin-layer chromatography (hexane/ethyl acetate 4:1). To the separation of the product, the catalyst was separated by an external magnet and the crude solid product was filtered and washed three times with ethanol (Figure 1B).

### 2.5 General procedure for the synthesis of 4,4′-(arylmethylene)bis(1H-pyrazole-5-ol) derivatives

3-methyl-1-phenyl-1H-pyrazole-5-ol (2 mmol), an aldehyde (1.0 mmol), and poly(aniline-*co*-melamine)@MnFe_2_O_4_ nanocomposite (0.05 g) were mixed, and the reaction was stirred at room temperature to finished. An external magnet was used to isolate the catalyst. The unpurified solid product was filtered, then refined using water and ethanol recrystallization (50:50) (Figure 1C).

### 2.6 General procedure for the synthesis of bis 4,4′-(arylmethylene)bis(1H-pyrazole-5-ol) derivatives

3-methyl-1-phenyl-1H-pyrazole-5-ol (2 mmol), an aldehyde (0.5 mmol), and poly(aniline-*co*-melamine)@MnFe_2_O_4_ nanocomposite (0.05 g) were mixed, and the reaction was stirred at room temperature to finished. An external magnet was used to isolate the catalyst. The crude solid product was filtered and washed three times with ethanol (Figure 1D).

### 2.7 Antioxidant activity

The antioxidant activity of 1,4-dihydropyrano[2,3-*c*]pyrazoles (4q, 4d, 4o, 4l), 4,4′-(arylmethylene)bis(1H-pyrazole-5-ol) (5i, 5j, 5q), bis 4,4'-(arylmethylene)bis(3-methyl-1H-pyrazol-5-ols) (6c) derivative was evaluated by DPPH (2,2-diphenyl-1-picrylhydrazyl) radical scavenger assy. Accordingly, derivatives were examined for their ability to the bleaching of the purple-colored ethanol solution DPPH. 100 mg of each sample was reacted with 1 ml of ethanolic DPPH at room temperature. Afterward, the absorbance of the reaction mixture was measured at 517 nm and the inhibition percentage was evaluated by the following formula (Ghahremanloo et al., 2022): DPPH inhibition (%) = A_b_-A_s_/A_b_ ×100.

### 2.8 *In-vitro* anticancer study

The anti-cancer activity of 1,4-dihydropyrano[2,3-*c*]pyrazoles (4q, 4d, 4o, 4l), 4,4′-(arylmethylene)bis(1H-pyrazole-5-ol) (5i, 5j, 5q), bis 4,4'-(arylmethylene)bis(3-methyl-1H-pyrazole-5-ols) (6c) derivative was investigated by MTT assay. Briefly, 3,000 cells (HCT116 as the human colon cancer cell line and L929 as the normal cell line) were seeded in 96 well culture plates. After attachment, cells were treated with 4q, 4d, 4o, 4l, 5i, 5j, 5j, 5q and 6c (12.5–200 ug/mL). In the time points of 24 and 48 h after treatment, MTT dye (0.5 mg/ml) was added to each well. Plates were incubated at 37°C for 4 h, the cultured medium was withdrawn and 100 µl of DMSO was added to each well. Plates were shaken for 20 min and their absorbance was read at 570 nm using a plate reader. The viability of treated cells was calculated by the following formula (Hu et al., 2019): Viability (%) (Absorbance of treated cells/Absorbance of control cells) × 100.

### 2.9 Characterization

Fourier transform infrared spectroscopy (FTIR, Bruker Tensor 27, Bremen, Germany), hydrogen and carbon nuclear magnetic resonance spectroscopy (^1^HNMR and ^13^CNMR, Bruker Avance DRX-400, Bremen, Germany) were employed for the chemical characterization of the products. The crystallinity and surface morphology of samples were investigated by X-ray diffraction (XRD, Shibuya-ku, Tokyo, Japan) and field emission scanning electron microscope (FESEM, Hitachi S4160 instrument, Japan), energy dispersive X-ray analysis (EDX, MFTIRA III, TESCAN, Czech Republic), respectively. Thermogravimetric analysis (TGA 209F3, NETZSCH, Selb, Germany) was utilized to test the thermal stability of samples.

## 3 Results and discussions

### 3.1 Characterization of polymer-based nanocatalyst

#### 3.1.1 Fourier transform infrared spectroscopy

**FIGURE 2:**
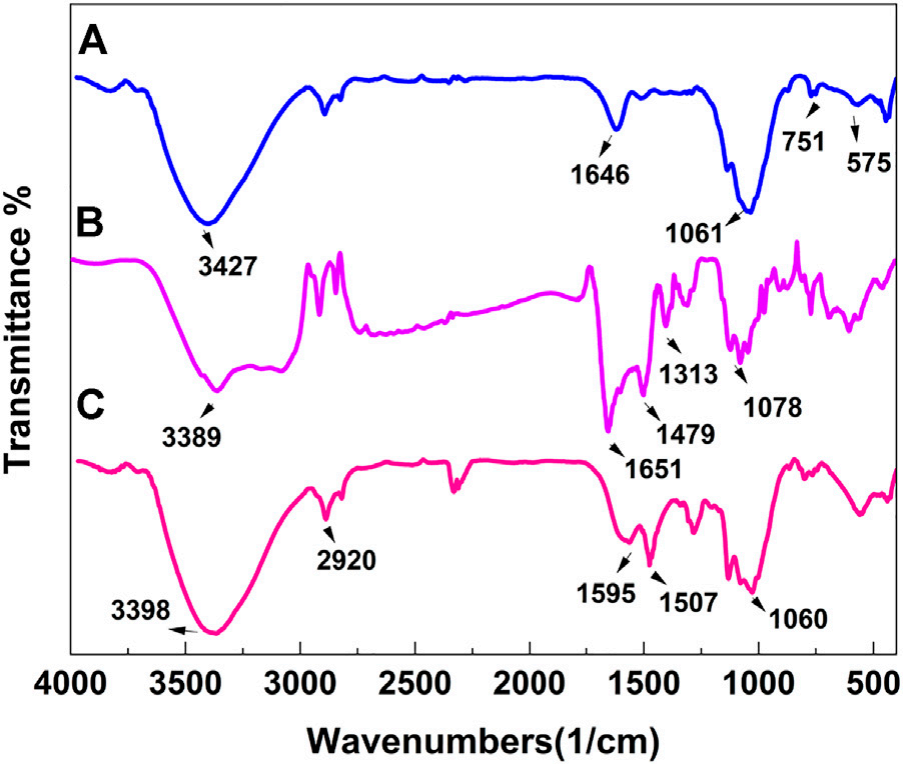
FTIR spectra of the prepared MnFe_2_O_4_ **(A)**, poly(aniline-*co*-melamine) **(B)**, and poly(aniline-*co*-melamine) @MnFe_2_O_4_ **(C)**

The FTIR absorption spectra of the prepared MnFe_2_O_4_ (a), poly(aniline-*co*-melamine) (b), poly(aniline-*co*-melamine)@MnFe_2_O_4_ (c) nanocomposite are shown in Figure 2. The MnFe_2_O_4_ spectrum exhibits two prominent absorption bands below 1,000 cm^−1^, which is a characteristic of ferrites. The distinctive bands at 449 and 573 cm^−1^ are caused by the metal-inherent oxygen’s stretching vibrations at the octahedral and tetrahedral sites, respectively (Zipare et al., 2015). The poly(aniline-*co*-melamine) (b) and poly(aniline-*co-*melamine)@MnFe_2_O_4_ (c) samples displayed very similar spectra. The stretching vibration of the NH_2_ and N-H groups is observed at 3,390 cm^−1^. The stretching modes of benzenoid amine and quinoid imine units, respectively, are associated with the two distinctive bands at around 1,500 and 1,600 cm^−1^. The vibrations of the C-H and C-N stretching bonds in the benzene ring are responsible for the peaks at 1,106 and 1,296 cm^−1^, respectively. The band corresponding to the bending vibrations of the C-H bond in the p-disubstituted benzene rings can be seen in the 797 cm^−1^ (Maroufi et al., 2020).

#### 3.1.2 X-ray diffraction

**FIGURE 3:**
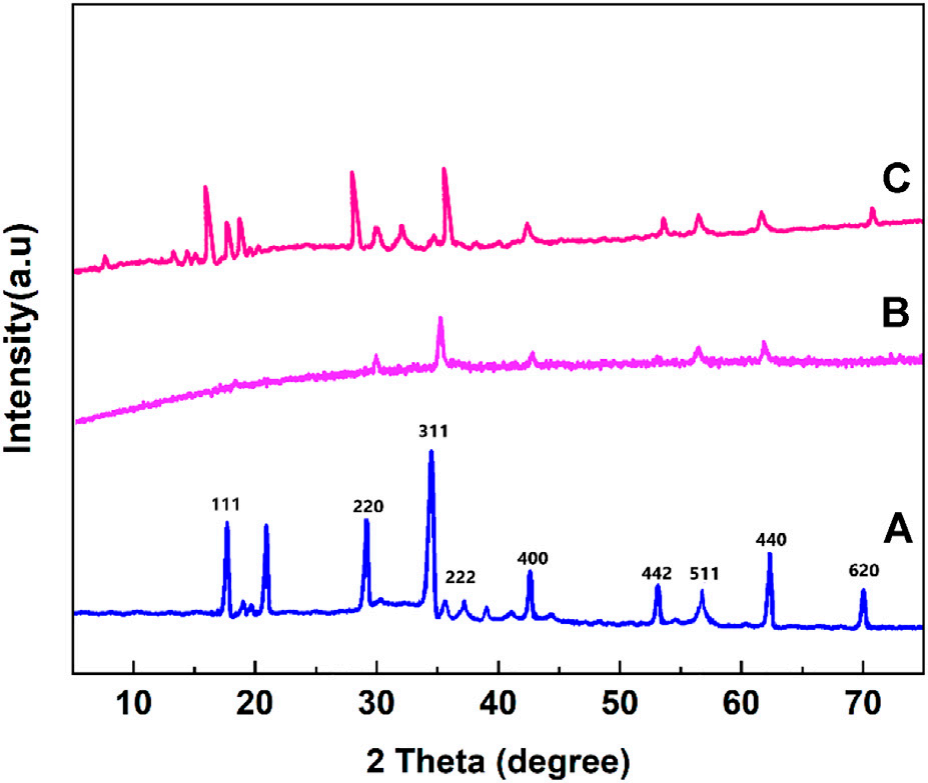
XRD patterns of the prepared MnFe_2_O_4_ **(A)**, poly(aniline-*co*-melamine) **(B)**, and poly(aniline-*co*-melamine) @MnFe_2_O_4_ **(C)**.

XRD patterns of the prepared MnFe_2_O_4_, poly(aniline-*co*-melamine), poly(aniline-*co*-melamine)@MnFe_2_O_4_ nanocomposite are shown in Figure 3. The XRD pattern of MnFe_2_O_4_ nanoparticles revealed a crystalline character. This pattern reveal the typical diffraction peaks of the MnFe_2_O_4_ nanoparticles at 18.10°, 30.60°, 36.15°, 43.65°, 54.10°, 57.65°, and 63.25° are attributable to the crystal planes of (111), (220), (311), (400), (422), (511), (440), and (620) respectively (Qin et al., 2021). The XRD patterns of the poly(aniline-*co*-melamine) (b) and (polyaniline-*co*-melamine)@MnFe_2_O_4_ nanocomposite (c) showed a semicrystalline nature. The existence of corresponding peaks of MnFe_2_O_4_ and poly(aniline-*co*-melamine) approved the successful preparation of the nanocomposite.

#### 3.1.3 Thermogravimetric analysis

**FIGURE 4:**
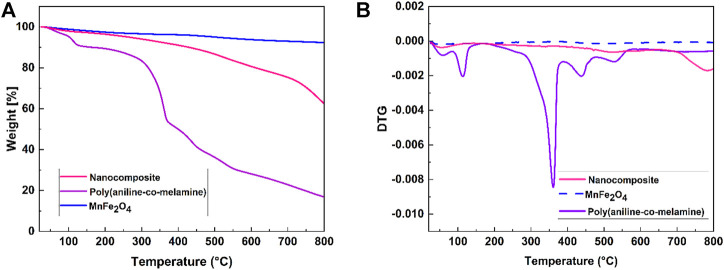
TGA **(A)**, and DTG thermograms **(B)** of the prepared MnFe_2_O_4_, poly(aniline-*co*-melamine), and poly(aniline-*co*-melamine) @MnFe_2_O_4_.

The TG/DTG curves of the MnFe_2_O_4_, poly(aniline-*co*-melamine), and poly(aniline-*co*-melamine)@MnFe_2_O_4_ nanocomposite are given in Figures 4A,B. The weight loss (below 150°C) observed in the TG curve of MnFe_2_O_4_ is related to the evaporation of adsorbed water on the surface of the nanoparticles. Three weight losses are observed in the TG curve of poly(aniline-*co*-melamine). The first step of degradation between 80°C and 200°C corresponded to theremoval of water. The second and third weight losses at 210°C and up to 800°C are related to the melamine breakdown and minor degradation of polymeric chains, respectively (Maroufi et al., 2020). The comparison of TG curves of poly(aniline-*co*-melamine)and poly(aniline-*co*-melamine)@MnFe_2_O_4_ nanocomposite showed that the stability of poly(aniline-*co*-melamine)@MnFe_2_O_4_ nanocomposite was higher than the poly(aniline-*co*-melamine).

#### 3.1.4 VSM

**FIGURE 5:**
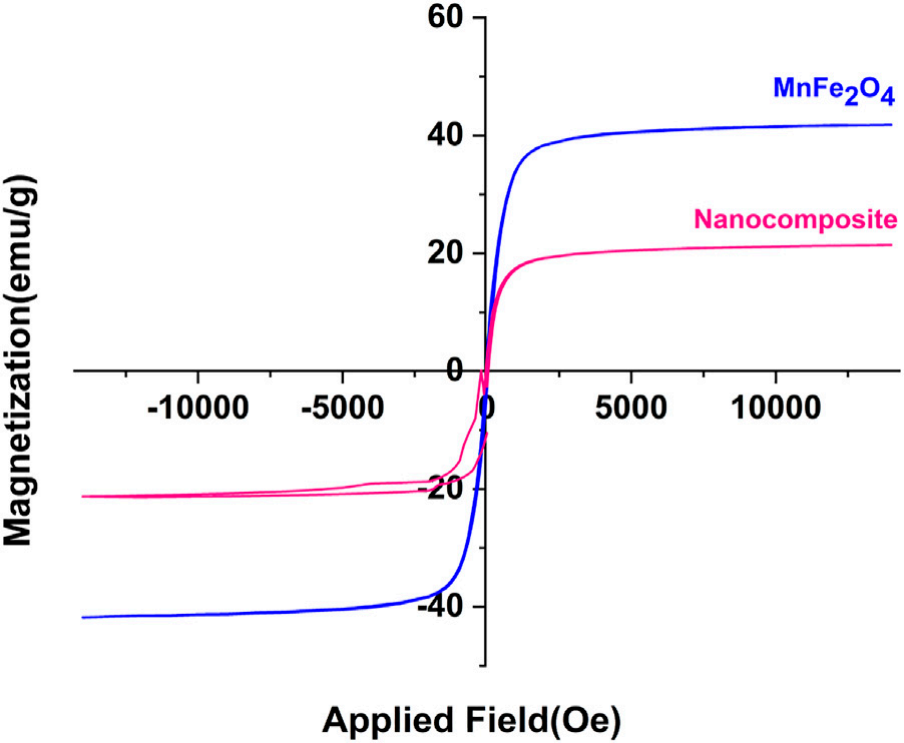
VSM curves of the prepared MnFe_2_O_4_ and poly(aniline-*co*-melamine)@MnFe_2_O_4_.

The VSM curves of the poly(aniline-*co*-melamine)@MnFe_2_O_4_ nanocomposite and MnFe_2_O_4_ are revealed in Figure 5. The VSM curves show that the magnetization saturation values of MnFe_2_O_4_ nanoparticles and poly(aniline-*co*-melamine)@MnFe_2_O_4_ were 21.28 and 41.81 emu/g, respectively. In addition, the magnetic property of the nanocomposite was lower than the MnFe_2_O_4_ nanoparticles. This could be related to covering the magnetic nanoparticles with poly(aniline-*co*-melamine) copolymer.

#### 3.1.5 UV-vis

**FIGURE 6:**
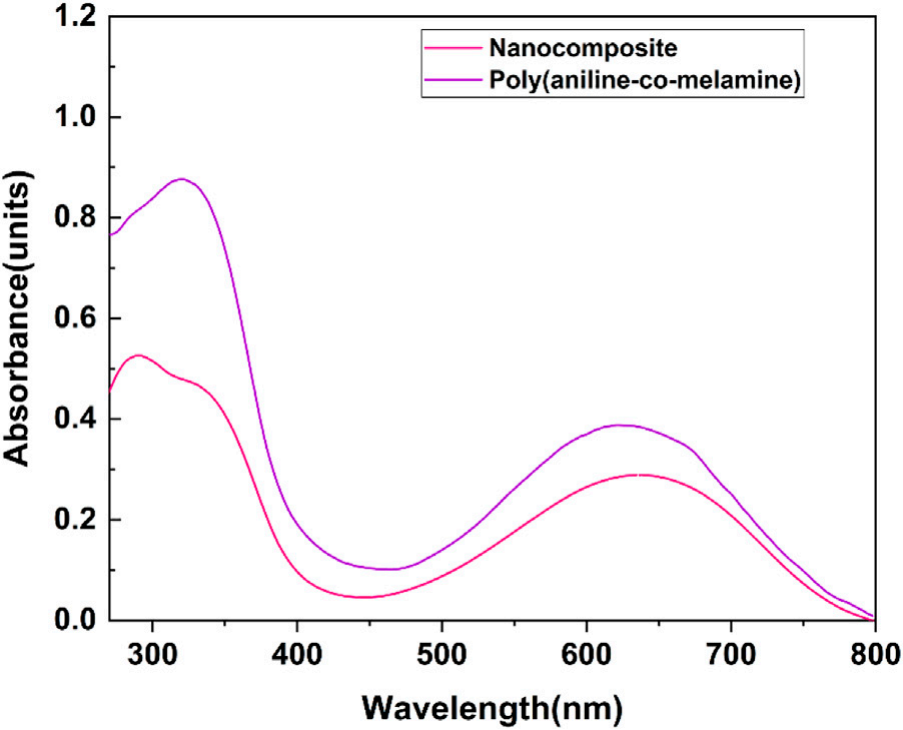
UV-vis absorption spectra of poly(aniline-*co*-melamine) and poly(aniline-*co*-melamine)@MnFe_2_O_4_ in DMSO solvent.

The UV–vis absorption spectra of poly(aniline-*co*-melamine), and poly(aniline-*co*-melamine)@MnFe_2_O_4_ are presented in Figure 6. A specific DMSO concentration was used to evaluate both samples. The UV–vis spectrum of poly(aniline-*co*-melamine) shows two characteristic absorption peaks assigned to the n-π* and π–π* electron transition within the benzenoid segments in the range of 300–400 nm. A charge transfer from the benzenoid rings to the quinoid rings causes the absorption peak at roughly 620 nm (Maroufi et al., 2020). The presence of MnFe_2_O_4_ in the nanocomposite leads to reducing in the solubility of the poly(aniline-*co*-melamine)@MnFe_2_O_4._ The UV–vis absorption spectrum of poly(aniline-*co*-melamine)@MnFe_2_O_4_ is weaker than the UV–vis spectrum of poly(aniline-*co*-melamine).

#### 3.1.6 Field emission scanning electron microscope

**FIGURE 7:**
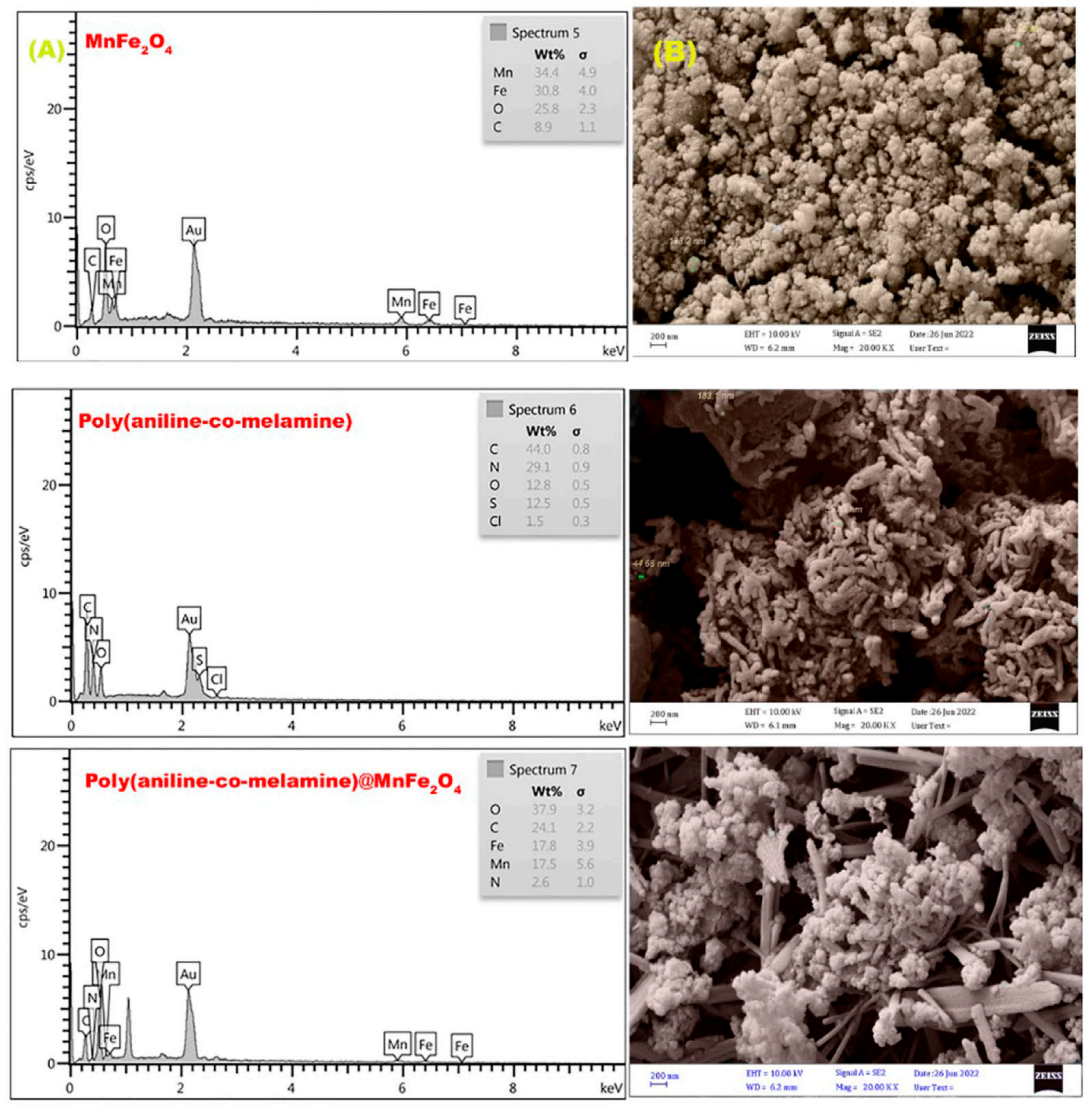
EDX spectra **(A)**, and FESEM micrographs **(B)** of the prepared MnFe_2_O_4_, poly(aniline-*co*-melamine), and poly(aniline-*co*-melamine) @MnFe_2_O_4_ nanocomposite.

Figure 7A displays the FESEM images of MnFe_2_O_4_, poly(aniline-*co*-melamine), and poly(aniline-*co*-melamine) @MnFe_2_O_4_ in magnifications of 200 nm. The average particle size for the of MnFe_2_O_4_ was 60 nm, The FESEM image of poly(aniline-*co*-melamine) showed rod-like structure with a diameter of 44–183 nm. The FESEM image of poly(aniline-*co*-melamine)@MnFe_2_O_4_ show the particle size decreased after the composite was prepared. The average particle size diameter was 50 nm. These findings revealed that The FESEM image of the nanocomposite was successfully prepared.

#### 3.1.7 Energy dispersive X-ray analysis

As shown in Figure 7B, the chemical composition of the produced MnFe_2_O_4_, poly(aniline-*co*-melamine), and poly(aniline-*co*-melamine) @MnFe_2_O_4_ was also determined using the EDXanalysis. The presence of various quantities of O, C, N, Mn, and Fe elements was proven by comparing spectra and tabular data. The occurrence of S and Cl in poly(aniline-*co*-melamine) is related to ammonium persulfate and HCl trapped in the polymer matrix.

### 3.2 Evaluation of the catalytic activity of poly(aniline-*co*-melamine)@MnFe_2_O_4_ nanocomposite through the synthesis of 1,4-dihydropyrano[2,3-*c*]pyrazole derivatives

To test the catalytic activity of poly(aniline-*co*-melamine)@Mn(Fe_2_O_4_), first the synthesis of 1,4-dihydropyrano[2,3-*c*]pyrazoles utilizing aromatic aldehyde, 3-methyl-1-phenyl-1H-pyrazole-5-ol, and malononitrile were carried out (Figure 1B).

The reaction conditions of benzaldehyde, 3-methyl-1-phenyl-1H-pyrazole-5-ol, and malononitrile in the presence of poly(aniline-*co*-melamine)@MnFe_2_O_4_ as a catalyst were optimized to get a higher yield. Following that, a solvent screening indicated that the reaction was more successful in water and ethanol. When benzaldehyde (1 mmol), and 3-methyl-1-phenyl-1*H*-pyrazole-5-ol (1 mmol) were reacted with malononitrile (1 mmol) in the presence of Poly(aniline-*co*-melamine)@MnFe_2_O_4_ (0.05 g) in Ethanol/H_2_O (1:1) at 40°C, the best results were attained (Table 1 entry 11). On the other hand, it is confirmed that the reaction requires a catalyst to continue and that the efficiency is not high in the absence of a catalyst (Table 1, entry 12). In this reaction model, the conversion rates were examined in the presence of poly(aniline-*co*-melamine) and MnFe_2_O_4_ (Table 1, entries 13, 14), the results showing that the best result has been achieved in poly(aniline-*co*-melamine) but the major problem of poly(aniline-*co*-melamine) as a catalyst is easy solubility in organic solvents, and therefore its isolation is difficult. The poly(aniline-*co*-melamine)@MnFe_2_O_4_ nanocomposite was consequently chosen as an efficient catalyst to carry out the reactions.

**TABLE 1:** Optimization of the three-component reaction of 3-methyl-1-phenyl-1*H*-pyrazole-5-ol, benzaldehyde, and malononitrile under various conditions.^a^

Entry	Solvent	Catalyst (g)	Temp/^°^C	Time/min	Yield%^b^
1	EtOH/H_2_O	Catalyst 0.04	40	35	85
2	H_2_O	Catalyst 0.04	40	60	50
3	THF	Catalyst 0.04	40	120	35
4	EtOH	Catalyst 0.04	40	60	60
5	CHCl_3_	Catalyst 0.04	40	120	45
6	Solvent-free	Catalyst 0.04	100	120	55
7	EtOH/H_2_O	Catalyst 0.04	60	30	65
8	EtOH/H_2_O	Catalyst 0.04	Reflux	60	60
9	EtOH/H_2_O	Catalyst 0.04	r.t	60	55
10	EtOH/H_2_O	Catalyst 0.03	40	50	80
11	**EtOH/H**_**2**_ **O**	**Catalyst 0.05**	**40**	**20**	**90**
11	EtOH/H_2_O	Catalyst 0.06	40	20	90
12	EtOH/H_2_O	—	40	120	50
13	EtOH/H_2_O	Poly(aniline-*co*-melamine) 0.05	40	15	90
14	EtOH/H_2_O	MnFe_2_O_4_ 0.05	40	60	50

^a^ Reaction conditions: aldehyde (1 mmol), 3-methyl-1-phenyl-1*H*-pyrazol-5-ol (1 mmol), and malononitrile (1 mmol).

^b^ Isolated yield.

The bold value row showed the best condition.

Obtained data from Table 1 showed that the catalytic activity of poly(aniline-*co*-melamine)@MnFe_2_O_4_ nanocomposite is higher compared to poly(aniline-*co*-melamine) and MnFe_2_O_4_. This result can be explained according to FESEM images. In the FESEM images particle size in the poly(aniline-*co*-melamine)@MnFe_2_O_4_ nanocomposite is smaller than the poly(aniline-*co*-melamine) and larger than the MnFe_2_O_4_ nanoparticles. In addition, the morphology of nanocomposite is different from MnFe_2_O_4_ nanoparticles and poly(aniline-co-melamine) which showed agglomerate particles with disordered rod-like structures. This observation showed that the presence of MnFe_2_O_4_ nanoparticles in the copolymer matrix led to decreasing the particle size and differencing in the morphology of the nanocomposite. It is well known that particle size and morphology are two important parameters that influence the catalytic performance of nanocatalysts (Kamalzare, 2022). Nanocatalysts with a high surface area could provide more active sites to less activation energy for catalytic reactions, and novel surface structures lead to product selectivity and catalytic efficacy. Thus, the higher catalytic activity of nanocomposite can be related to the high volume-to-surface ratio and different morphology of the nanocomposite.

The reaction with several aromatic aldehydes to yield a wide range of 1, 4-dihydropyrano[2,3-*c*]pyrazoles were examined in the terms of substrate scope (Table 2). The presence of poly(aniline-*co-*melamine)@MnFe_2_O_4_ revealed that this reaction is generally tolerable to a wide range of benzaldehyde derivatives comprising electron-withdrawing and electron-donating groups (0.05 g). Calculation of turnover number (TON) and turnover frequencies (TOF) provides a comprehensive way to make comparisons based on a per active site basis. These values have been calculated from the EDX and TGA data. The results are shown in Table 2 (Al-Hasani et al., 2017; Molaei and Javanshir, 2018).

**TABLE 2:** Synthesis of 1,4-dihydropyrano[2,3-*c*]pyrazoles by poly(aniline-*co*-melamine)@MnFe_2_O_4_ and benzaldehyde derivatives.^a^

Entry	Product	Code	Time. min	Yield%	TON	TOF X 10^−3^(h^−1^)	M.P/°C		Ref
							Observed	Reported	
1	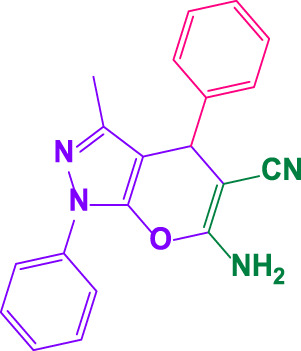	**4a**	20	90	3,333	10.1	169–172	168–169	Vafajoo et al. (2015)
2	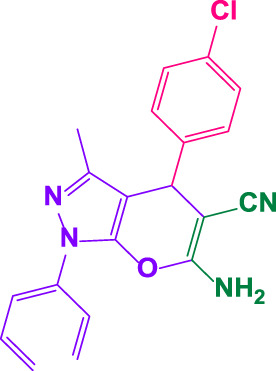	**4b**	15	95	3,518	14.0	172–173	174–176	Vafajoo et al. (2015)
3	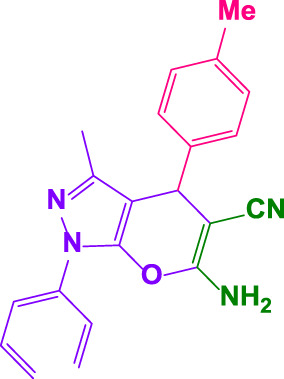	**4c**	25	91	3,370	8.2	173–175	172–173	Vafajoo et al. (2015)
4	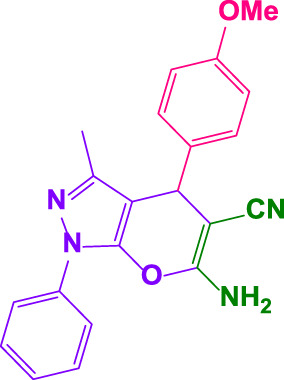	**4d**	15	90	3,333	13.3	170–173	172–174	Vafajoo et al. (2015)
5	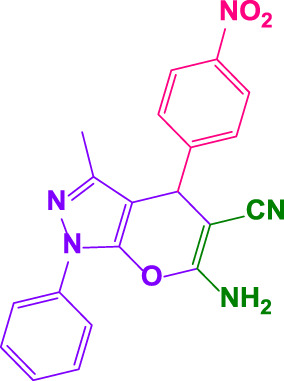	**4e**	**10**	93	3,444	20.7	192–193	190–195	Vafajoo et al. (2015)
6	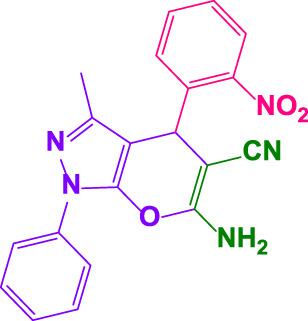	**4f**	15	92	3,407	13.6	189–191	190–192	Vafajoo et al. (2015)
7	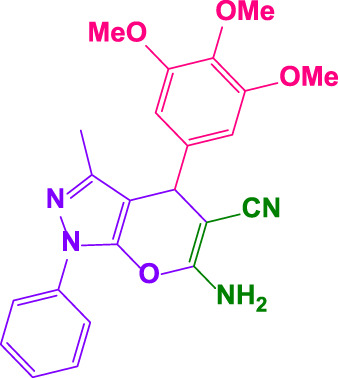	**4g**	20	89	3,269	9.9	202–204	200–203	Jasim et al. (2022)
8	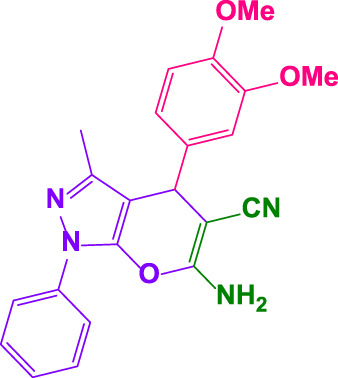	**4h**	25	90	3,333	8.1	196–198	193–195	Vafajoo et al. (2015)
9	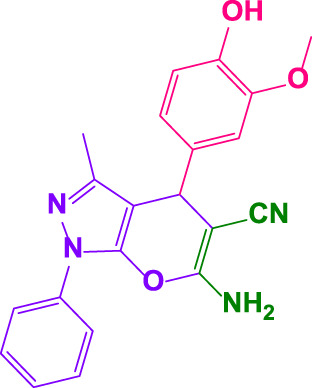	**4i**	25	93	3,444	8.4	191–193	193–195	Zolfigol et al. (2016)
10	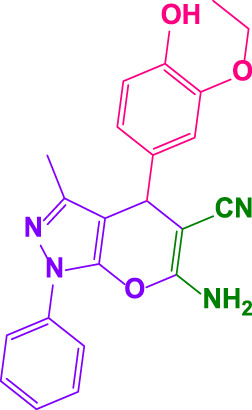	**4j**	25	92	3,407	8.3	172–173	169–171	Vafajoo et al. (2015)
11	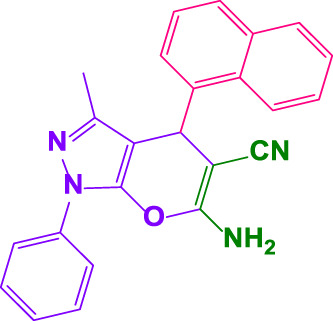	**4k**	15	95	3,518	14.0	240–242	244–245	Zolfigol et al. (2016)
12	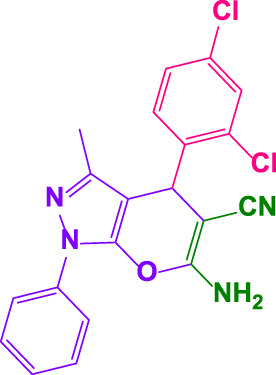	**4l**	10	96	3,555	21.04	179–181	180–182	Vafajoo et al. (2015)
13	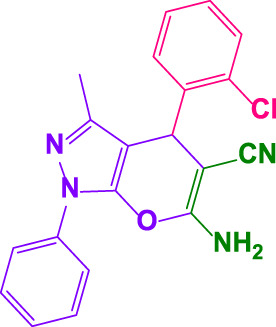	**4m**	15	90	3,333	13.3	147–149	145–146	Vafajoo et al. (2015)
14	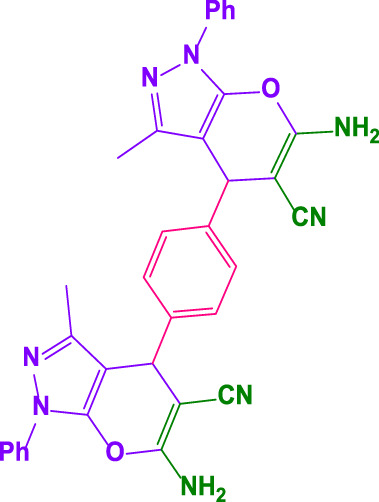	**4n**	30	90	3,333	6.6	233–235	236–238	Vafajoo et al. (2015)
15	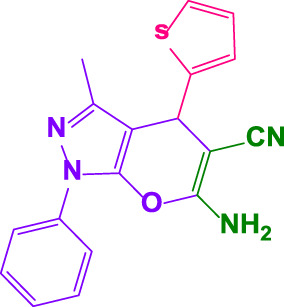	**4o**	30	90	3,333	6.6	168–170	167–168	Zolfigol et al. (2016)
16	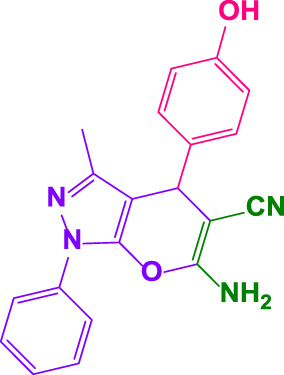	**4p**	30	94	3,481	6.9	212–213	211–213	Vafajoo et al. (2015)
17	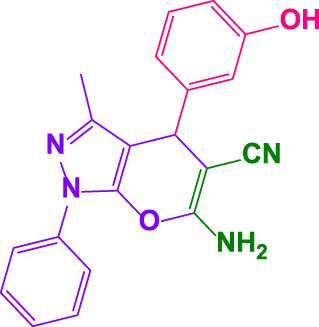	**4q**	35	90	3,333	5.7	208–209		

^a^ Reaction conditions: aldehyde (1 mmol), 3-methyl-1-phenyl-1*H*-pyrazol-5-ol (1 mmol), and malononitrile (1 mmol), 5 ml water/EtOH (1:1) and poly(aniline-*co*-melamine)@MnFe_2_O_4_ (0.05) at 40°C.

The bold value row showed the best condition.

#### 3.2.1 Suggested mechanism

**FIGURE 8:**
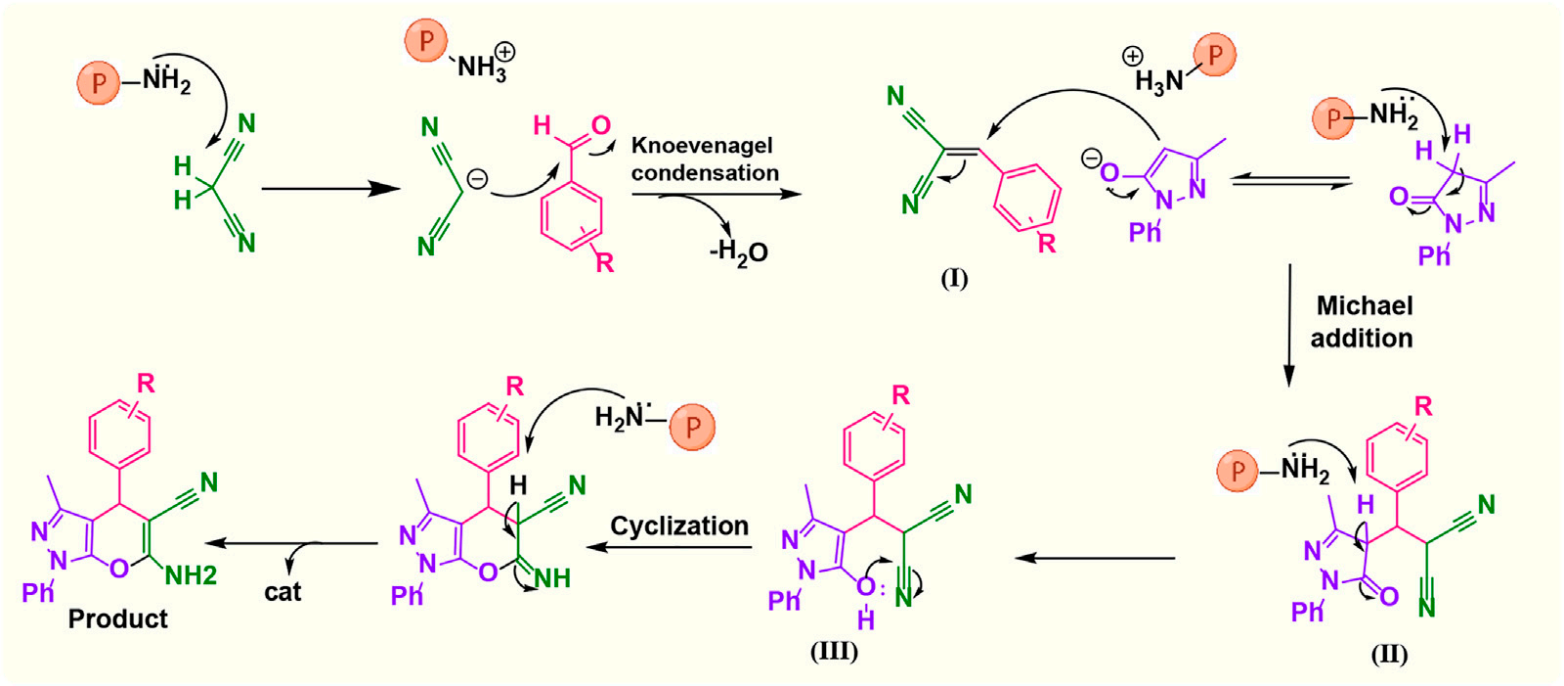
Suggested mechanistic scheme for the synthesis of 1,4-dihydropyrano[2,3-*c*]pyrazole catalyzed by poly(aniline-*co*-melamine)@Mn(Fe_2_O_4_). ℗ represents a polymer-based catalyst.

A possible mechanism (Figure 8) involves the initial activation of malononitrile using the catalyst and then attacking the carbonyl group of the aldehyde. By removing one water molecule, intermediate (I) is prepared (Knoevenagel condensation). The reaction is followed by deprotonation of 3-methyl-1-phenyl-1*H*-pyrazole-5-ol by a catalyst. Then, Michael’s addition of 3-methyl-1-phenyl-1*H*-pyrazole-5-ol to intermediate (I) affords (II). Intermediate (II) was converted to (III) *via* tautomerization, and the product is obtained by cyclization (Hajizadeh and Maleki, 2018).

The poly(aniline-co-melamine)@MnFe_2_O_4_ was employed also as a catalyst in the synthesis of bis pyrazolone. We investigated the synthesis of bispyrazolones using aromatic aldehyde (1 mmol) and 3-methyl-1-phenyl-1*H*-pyrazole-5-ol (2 mmol) (Figure 1C). In order to optimize this reaction, benzaldehyde and 3-methyl-1-phenyl-1*H*-pyrazole-5-ol were selected as starting materials, and the effects of temperature, amount of catalyst, solvent, etc., were investigated. The influence of varied quantities of catalysts on the reaction result was examined. Among different catalyst loadings (e.g., 0.04, 0.05, and 0.06 g), the catalyst based on poly(aniline-*co*-melamine)@MnFe_2_O_4_ (0.05 g) was selected as the most effective amount. Following that, it was investigated how the solvent affected the reaction’s result. According to the data, ethanol was the best solvent for this kind of domino reaction. The findings of the subsequent investigation into the impact of temperature showed that room temperature conditions produced the product with the highest yield (Table 3).

**TABLE 3:** Optimization of the reaction of 3-methyl-1-phenyl-1*H*-pyrazol-5-ol, and benzaldehyde, under various conditions^a^.

Entry	Solvent	Catalyst	Temp/^°^C	Time/min	Yield%^b^
1	EtOH/H_2_O	0.05	50	30	70
2	H_2_O	0.05	50	45	60
3	THF	0.05	50	100	30
4	EtOH	0.05	50	30	80
5	CHCl_3_	0.05	50	100	50
6	Solvent-free	0.05	50	100	70
7	EtOH	0.05	80	30	60
8	EtOH	0.05	r.t	20	95
9	EtOH	0.04	r.t	35	85
10	EtOH	0.07	r.t	20	96

^a^ Reaction conditions: aldehyde (1 mmol), 3-methyl-1-phenyl-1*H*-pyrazol-5-ol (2 mmol).

^b^ Isolated yield.

Different electron-withdrawing or electron-donating groups, such as OCH_3_, NO_2_, and CH_3_, were chosen to react with 3-Methyl-1*H*-phenyl-5-pyrazolone at the most favorable reaction conditions in order to examine the range and generality of this technique. Table 4 provides a summary of the findings. Different aromatic aldehydes containing both electron-withdrawing and electron-donating groups, including OH, OCH_3_, NO_2_, and CH_3_, generated their respective derivatives in good to exceptional yields, according to the experimental data. The calculation of turnover number (TON) and turnover frequencies (TOF) of 4,4′-(arylmethylene)bis(1*H*-pyrazole-5-ol) derivatives is shown in Table 4.

**TABLE 4:** Synthesis of 4,4′-(arylmethylene)bis(1*H*-pyrazole-5-ol) derivatives.^a^

Entry	Product	Code	Time (Min.)	Yield%	TON	TOF X 10^−3^(h^−1^)	M.p/°C		Ref
							Observed	Reported	
1	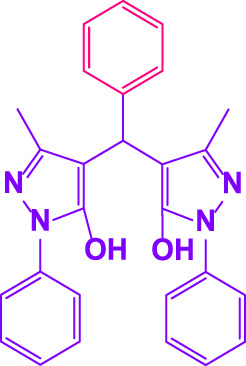	**5a**	20	95	3,518	10.6	171–173	173–174	Jahanshahi and Akhlaghinia, (2017)
2	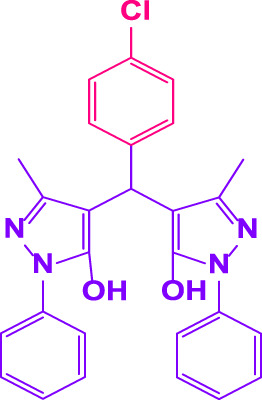	**5b**	15	96	3,555	14.2	212–215	205–207	Jahanshahi and Akhlaghinia, (2017)
3	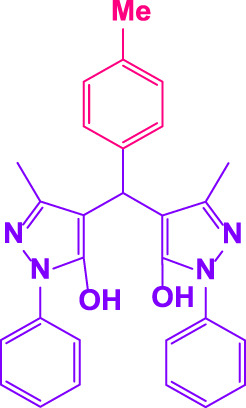	**5c**	25	93	3,444	8.4	203–205	202–204	Jahanshahi and Akhlaghinia, (2017)
4	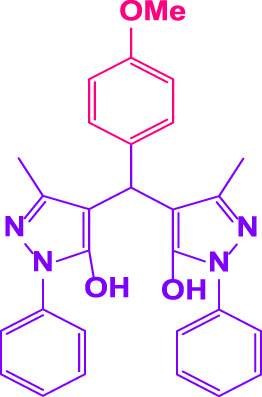	**5d**	20	96	3,555	10.7	173–175	172–174	Jahanshahi and Akhlaghinia, (2017)
5	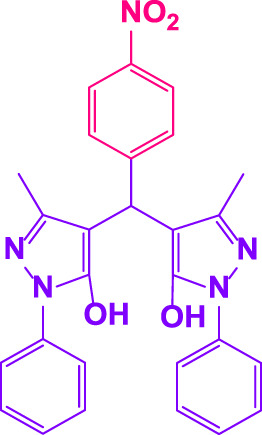	**5e**	**10**	96	3,555	22.2	223–225	230–232	Zolfigol et al. (2015)
6	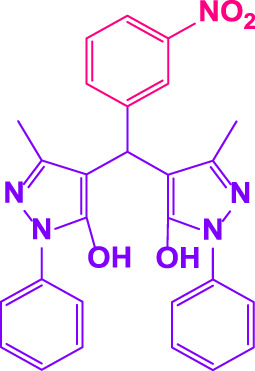	**5f**	15	92	3,407	13.6	150–152	148–149	Jahanshahi and Akhlaghinia, (2017)
7	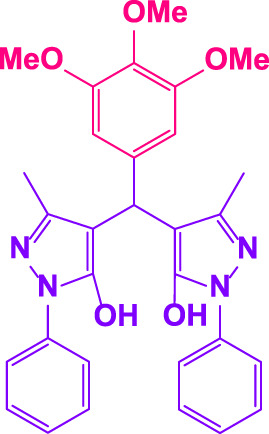	**5g**	30	95	3,518	7.0	195–197	194–196	Shabalala et al. (2020)
8	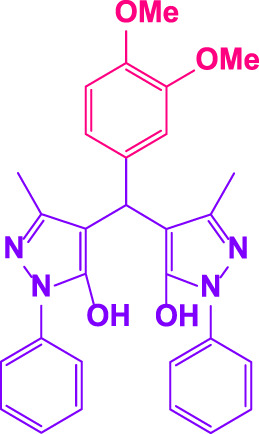	**5h**	30	94	3,481	6.9	155–157	153–155	Shabalala et al. (2020)
9	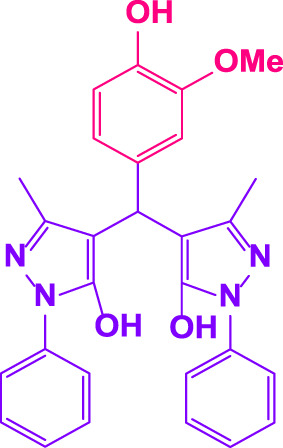	**5i**	25	96	3,555	8.6	206–208	205–207	Zolfigol et al. (2015)
10	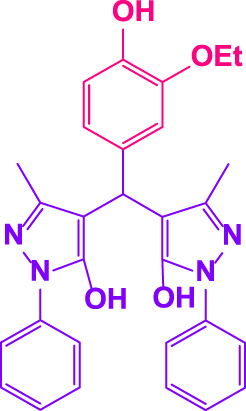	**5j**	30	92	3,407	6.8	205–207		
11	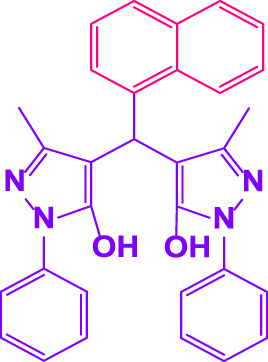	**5k**	20	90	3,333	10.1	212–214	210–212	Jahanshahi and Akhlaghinia, (2017)
12	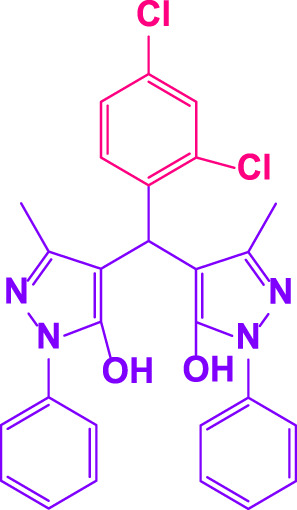	**5l**	10	95	3,518	21.9	226–228	227–229	Jahanshahi and Akhlaghinia, (2017)
13	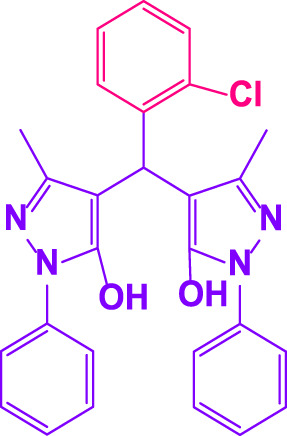	**5m**	15	94	3,481	13.9	233–235	235–237	Jahanshahi and Akhlaghinia, (2017)
14	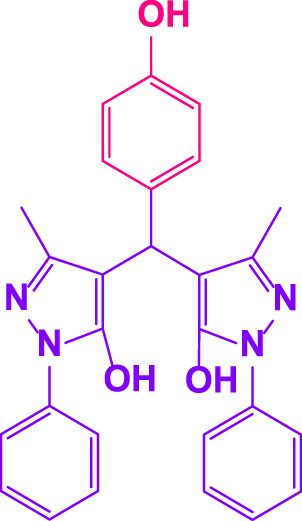	**5n**	30	90	3,333	6.6	159–161	159–160	Jahanshahi and Akhlaghinia, (2017)
15	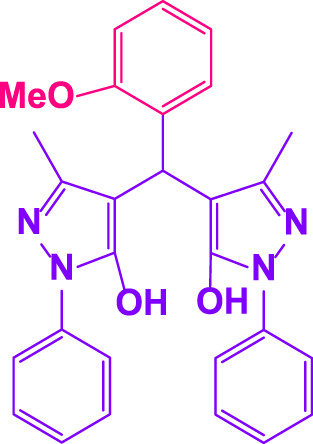	**5o**	25	92	3,407	10.32	214–215	212–213	Zolfigol et al. (2015)
16	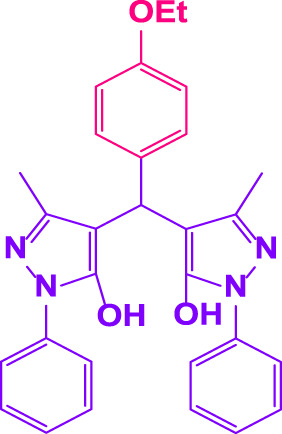	**5P**	30	90	3,333	6.6	190–191	185–188	Hasaninejad et al. (2011)
17	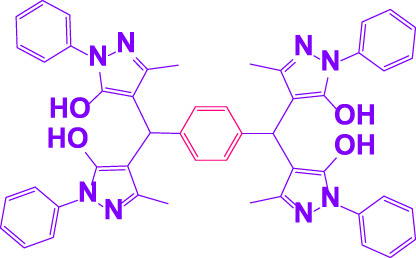	**5q**	70	94	3,481	3.1	194–196	193–193	Shabalala et al. (2020)

^a^ Reaction conditions: aldehyde (1 mmol), 3-methyl-1-phenyl-1*H*-pyrazol-5-ol (2 mmol), and EtOH (5 ml) and Poly(aniline-*co*-melamine)@MnFe_2_O_4_ (0.05) at room temperature conditions.

The bold value row showed the best condition.

#### 3.2.2 Suggested mechanism

**FIGURE 9:**
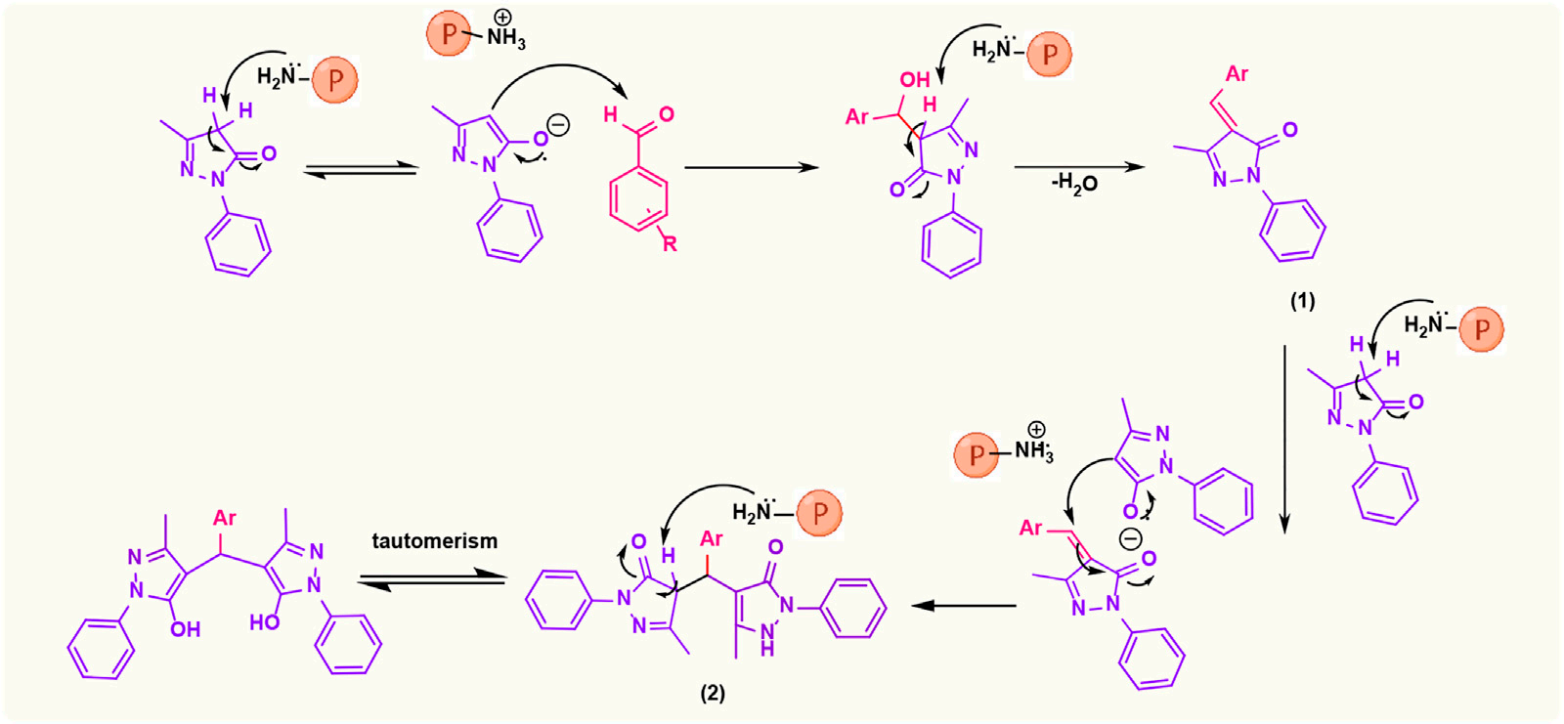
Suggested mechanistic scheme for the synthesis of 4,4′-(arylmethylene)bis(1*H*-pyrazole-5-ol) derivatives catalyzed by poly(aniline-*co*-melamine)@Mn(Fe_2_O_4_). ℗ represents polymer-based catalyst.

In the comparison of catalytic activity of poly(aniline-*co*-melamine) with nanoparticle MnFe_2_O_4_ in Table 1, the result showed that poly(aniline-*co*-melamine) is more active than MnFe_2_O_4_. Therefore, it seems that the amine group on the polymer will be the active site of the catalyst. A possible mechanism showed in (Figure 9). The initial deprotonation of 3-methyl-1-phenyl-1H-pyrazole-5-ol by amino groups in catalyst and then Knoevenagel condensation on the carbonyl group of the aldehyde that activated by ammonium ion. Then by removing H_2_O, intermediate (I) is prepared. The reaction is followed by deprotonation of 3-methyl-1-phenyl-1H-pyrazole-5-ol, then Michael’s addition of the resulted anion to intermediate 1 affords 2, and the product is obtained by tautomerization.

**FIGURE 10:**
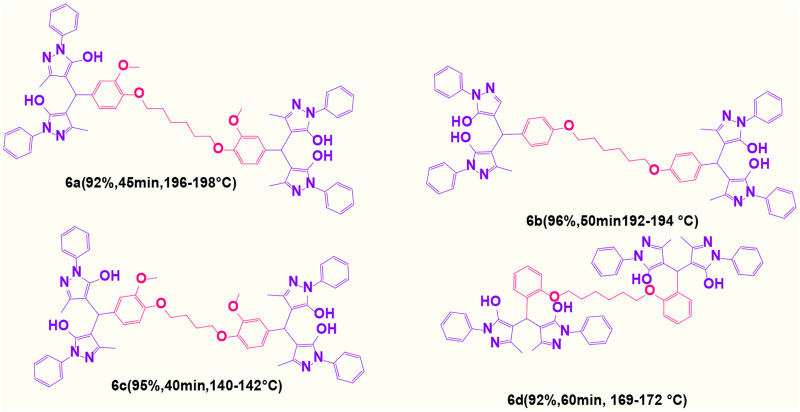
The structure of bis-4,4'-(arylmethylene)-bis-(3-methyl-1*H*-pyrazole-5-ols).

Here, it is described how to make the alkylene-bridging bis 4,4'-(arylmethylene)bis(3-methyl-1*H*-pyrazole-5-ols) derivative *via* a simple multicomponent reaction. To create a bifunctional aldehyde, dibromohexane (1 eq) and 2-hydroxybenzaldehyde (2 eq) were mixed. In the presence of poly(aniline-*co*-melamine)@MnFe_2_O_4_ as a catalyst, the resulting dialdehyde was condensed with the proper quantities of 3-methyl-1-phenyl-5-pyrazolone to produce bis 4,4'-(arylmethylene)bis (3-methyl-1*H*-pyrazole-5-ols) (Figure 10).

#### 3.2.3 Recovery

**FIGURE 11:**
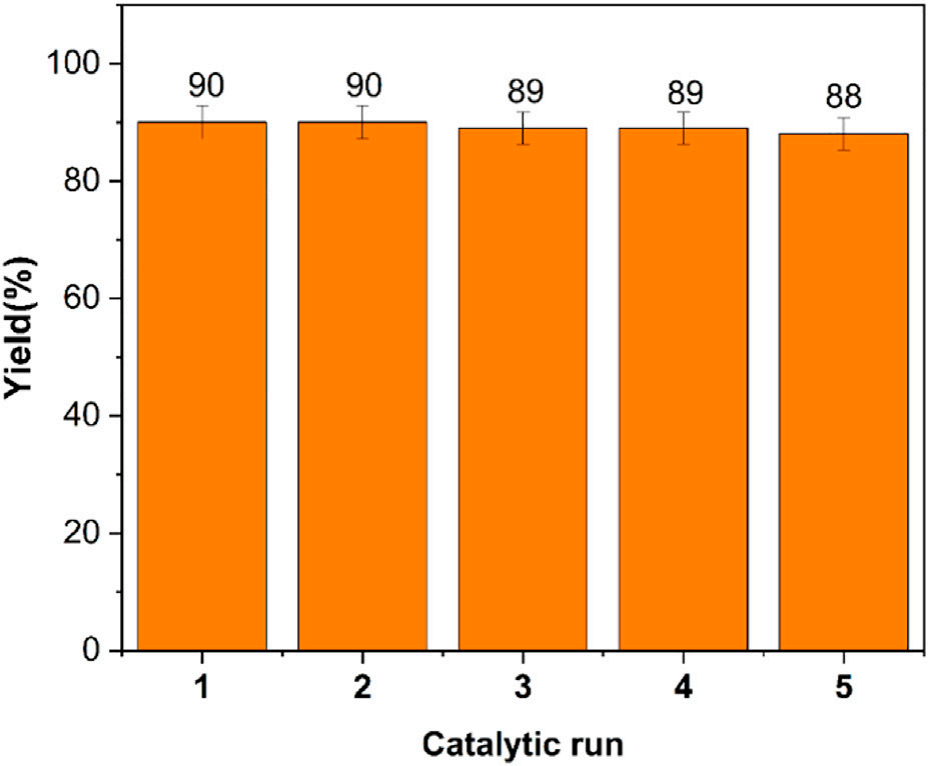
Reusability of poly(aniline-*co*-melamine)@MnFe_2_O_4_ in the synthesis 6- mino-3-methyl-1,4-diphenyl-1,4-dihydropyrano[2,3-*c*]pyrazole-5-carbonitrile (4a).

The possibility of reusing and recycling the catalyst for the production of 6-mino-3-methyl-1,4-diphenyl-1,4-dihydropyrano[2,3-*c*]pyrazole-5-carbonitrile (4a) was investigated. The catalyst was recovered after conducting the reaction of benzaldehyde, 3-methyl-1-phenyl-1*H*-pyrazole-5-ol, and malononitrile at the conditions specified in the experimental section. The catalyst was separated by an external magnet after adding hot ethanol to the reaction mixture. The catalyst was rinsed in ethanol (5 ml) and dried at low pressure. The catalyst was then re-fed into the reaction, as seen in Figure 11, where it continued to display good activity for the fifth time.

#### 3.2.4 Antioxidant activity

**FIGURE 12:**
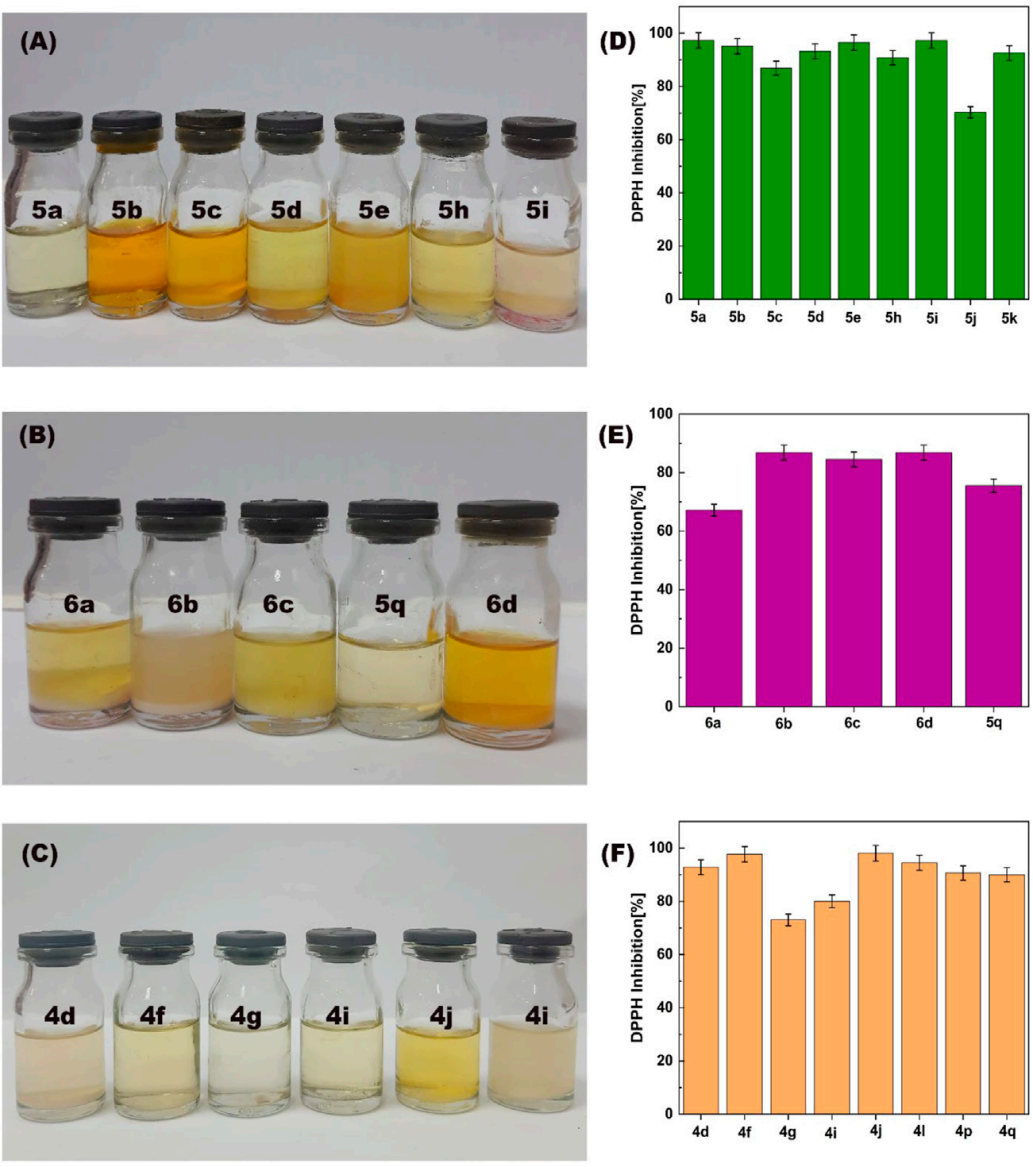
The photographs **(A–C)** and the histograms **(D–F)** of the antioxidant activity of synthesized of 1,4-dihydropyrano[2,3-*c*]pyrazoles (4q, 4f, 4g, 4i, 4d, and 4l), 4,4′-(arylmethylene)bis(1H-pyrazole-5-ol) (5a, 5b, 5c, 5d, 5e, 5h, 5i and 5q), bis 4,4'-(arylmethylene)bis(3-methyl-1H-pyrazol-5-ols) (6a, 6b, 6c, and 6d) derivatives.

The antioxidant activity of synthesized 4,4′-(arylmethylene)bis(1*H*-pyrazole-5-ols), bis 4,4′(arylmethylene) bis(3-methyl-1*H*-pyrazole-5-ols) and 1,4-dihydropyrano[2,3-*c*]pyrazoles were investigated using the 2,2-diphenyl-1-picrylhydrazyl (DPPH) scavenging model (Figure 12). The antioxidant activity of 22 synthesized derivatives was compared with ascorbic acid (vitamin c). Results showed that the antioxidant activity of synthesized derivatives after 60 min in an ethanolic solution of DPPH was between 70% and 98%. In addition, the antioxidant activity of ascorbic acid as a standard model was reported 96% (Janani et al., 2020) (Janani et al., 2020). These results showed that the synthesized derivatives can have good potential to be used in medicines or supplements with antioxidant properties.

#### 3.2.5 *In-*vitro anticancer study

**FIGURE 13:**
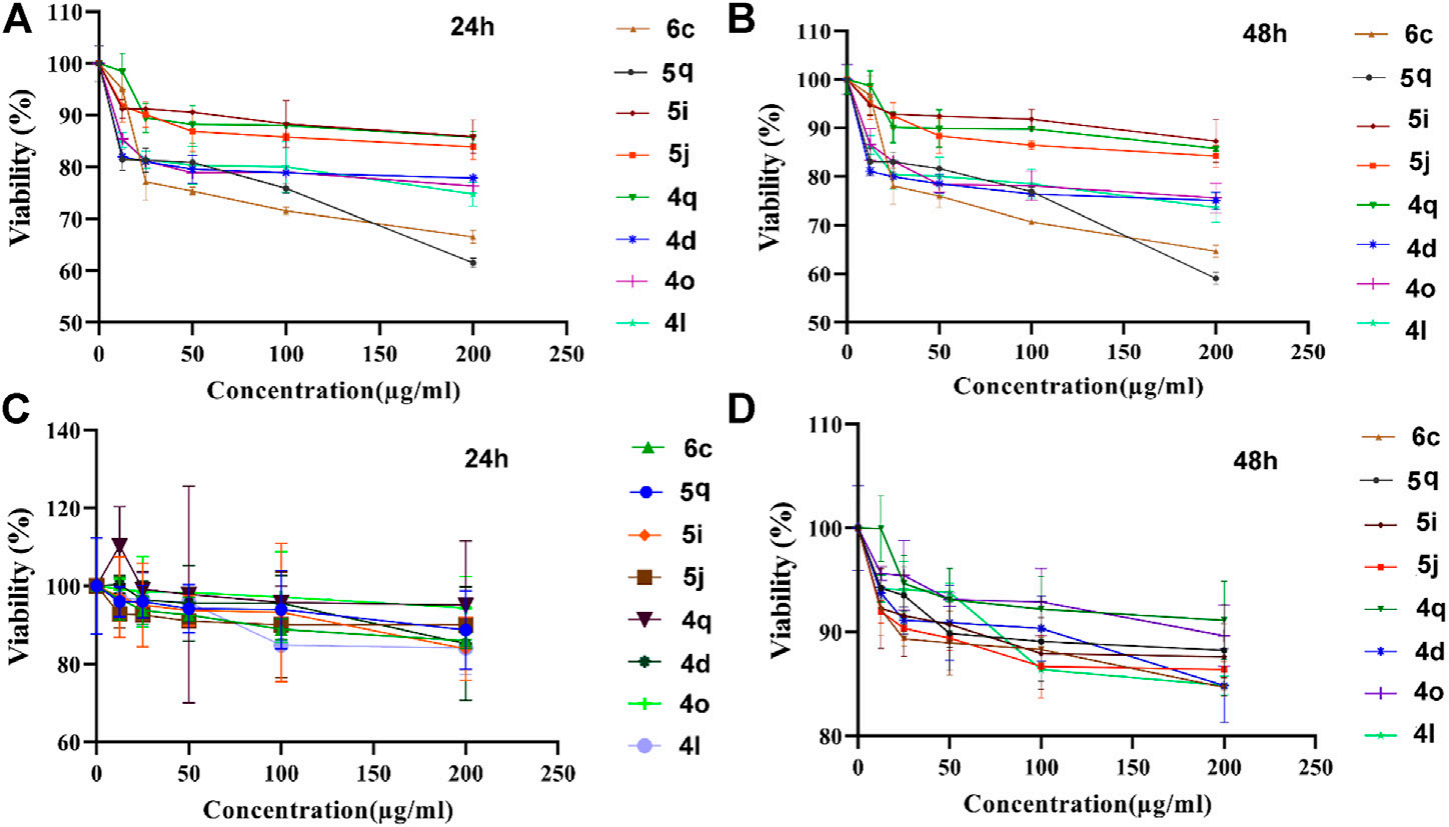
Anticancer activity of 1,4-dihydropyrano[2,3-*c*]pyrazoles (4q, 4d, 4o, 4l), 4,4′-(arylmethylene)bis(1H-pyrazole-5-ol) (5i, 5j, 5q), bis 4,4'-(arylmethylene)bis(3-methyl-1H-pyrazol-5-ols) (6c) derivatives against HCT116 **(A,B)** and L929 **(C,D)** cell lines.

Anticancer study was evaluated against colon cancer (HCT116) and normal (L929) cell lines within 24–48 h and results were reported as the percent growth of the treated cells when compared to the untreated control cells (Figure 13).

After 24 h of exposure to colon cancer cells (Figure 13A) 6c and 4q samples showed significant toxicity in 25 µg/ml and higher doses. Other samples (5i, 5j, 5q, 4d, 4o, and 4l) had toxicity in 12.5 µg/ml and higher doses. After 48 h (Figure 13B), the 4q sample showed significant toxicity in 50 µg/ml and higher doses. On the other hand, 6c, 5i, and 5j samples had significant toxicity in 25 µg/ml and higher doses. Other samples (5q, 4l, 4o, and 4d) showed considerable toxicity in 12.5 µg/ml and higher doses. These results show that the tested dervativies have good potential for destroying cancer cells and can be used in drug compounds.

For better comparison, the normal cell line was also exposed to tested samples and results showed that after 24 h (Figure 13C), no considerable toxicity was observed for 25 µg/ml and lower doses for all samples. 5j sample was toxic in 50 µg/ml and higher doses. In addition, 6c and 4l samples showed significant toxicity in 100 µg/ml and higher doses. 5q, 5i, and 4d samples were toxic only in 200 µg/ml concentration. 4q and 4o samples did not cause toxicity in any of the applied concentrations.

After 48 h (Figure 13D), no considerable toxicity was observed in the 12.5 µg/ml concentration. 6c, 5i, 5j, and 4d samples showed significant toxicity in 25 µg/ml and higher doses. 5q and 4q samples showed significant toxicity in 50 µg/ml and higher doses. 4l sample was toxic in 100 µg/ml and higher doses. 4o samples were toxic only in 200 µg/ml concentration.

Results showed that the most effective anticancer agents were 5q and 6c samples which lead to ∼30%–40% lethality in the highest applied dose (200 μg/ml). This concentration is relatively higher than conventional anticancer drugs. Two standard chemotherapeutic drugs commonly used in the treatment of colorectal cancer are 5-Fluorouracil (5-FU), and oxaliplatin (OXA) (Manoochehri et al., 2022). The IC50 for 5-FU in different colorectal cancer cell lines including HCT116 (used in this study) reported in a range between 0.6 and 4 μg/ml (Varghese et al., 2019). In a study performed by Yang et al. (2019), IC50 for 5-FU resistant HCT116 and HT29 colorectal cancer cell lines was 12.5 ± 1.4 μg/ml and 11.5 ± 1.5 μg/ml, respectively. They showed that IC50 of oxaliplatin in parental HCT-116 and OXA-resistant HCT116 cells was 9.18 and 70.01 μg/ml, respectively (Yang et al., 2019). However, chemical anticancer drugs have considerable toxicity against body normal cells owing to a lack of specificity (Sarfjoo et al., 2022), but 5q sample dose does not have considerable toxicity against normal cells in lower than 200 μg/ml doses in time points of 24 h.

## 4 Conclusion

A magnetic poly(aniline-*co*-melamine)@MnFe_2_O_4_ nanocomposite was prepared from poly(aniline-*co*-melamine) and manganese ferrite nanoparticles in two steps and was used as a catalyst in the synthesis of 1,4-dihydropyrano[2,3-*c*]pyrazoles, and 4,4′-(arylmethylene)bis(1*H*-pyrazole-5-ols) *via* one-pot, three-component condensation. FESEM image of nanocomposite showed disordered rod-like structures with an average particle size of 50 nm. In addition, TGA analysis showed high thermal stability for magnetic nanocomposite. The poly(aniline-*co*-melamine)@MnFe_2_O_4_ nanocatalyst showed great catalytic performance in the synthesis of 1,4-dihydropyrano[2,3-*c*]pyrazoles, 4,4′-(arylmethylene)bis(1*H*-pyrazole-5-ols) and new alkylene bridging bis 4,4′-(arylmethylene)bis(1*H*-pyrazole-5-ols) derivatives. The maximum yield of the 1,4-dihydropyrano[2,3-c]pyrazoles, 4,4′-(arylmethylene)bis(1H-pyrazole-5-ols)and 4,4′-(arylmethylene)bis(1H-pyrazole-5-ols) with 0.05 g of poly(aniline-co-melamine)@MnFe_2_O_4_ as nanocatalyst were obtained 95% in 15 min, 96% in 15 min, and 96% in 50 min, respectively.

The maximum yield of the 1,4-dihydropyrano[2,3-c]pyrazoles with 0.05 g of poly(aniline-comelamine)@MnFe2O4 at 40°C in EtOH/ water as a green solvent and mild conditions were obtained at 95 % in 15 min,. Also, tthe maximum yield of the 4,4′-(arylmethylene)bis(1H-pyrazole-5-ols) with 0.05 g of catalyst at r.t conditions in EtOH was obtained at 96 % in 15 min. and the maximum yield of the bis 4,4′-(arylmethylene)bis(1H-pyrazole-5-ols) with 0.05 g of catalyst at r.t conditions in EtOH was obtained at 96 % in 50 min. . Moreover, the poly(aniline-*co*-melamine)@MnFe_2_O_4_ nanocatalyst was separated easily from the reaction mixture by an external magnet. The catalyst could be reused at least five times, without a substantial loss of catalytic activity. The presence of amine and hydroxyl groups in the produced 1,4-dihydropyrano[2,3-*c*]pyrazoles and 4,4′-(arylmethylene)bis(1*H*-pyrazole-5-ols) resulted in antioxidant activity ranging from 70% to 98%. In addition, 4,4′-(arylmethylene)bis(1H-pyrazole-5-ol) derivatives and 1,4-dihydropyrano[2,3-*c*]pyrazoles showed good potential for destroying colon cancer cell line.

### 4.1 Spectroscopic data

#### 4.1.1 6- mino-3-methyl-1,4-diphenyl-1,4-dihydropyrano[2,3-c]pyrazole-5-carbonitrile (4a)

White powder, m. p. 169°C–172°C (Rep. 168°C–169°C) (Vafajoo et al., 2015); FTIR (KBr) υ_max_/cm^−1^: 3,447, 3,321, 2,921, 2199,1660, 1,560; ^1^H NMR (300 MHz, DMSO-*d*_*6*_): *δ* = 1.78 (s, 3H, CH_3_), 4.68 (s, 1H, benzilic), 7.23-7.35 (m, 8H, aromatic), 7.50 (t, 2H, *J =* 7.5* *Hz, aromatic),7.80 (d, 2H, *J* = *7.8* Hz, aromatic) ppm (Supplementary Figure S1).

#### 4.1.2 6-amino-4-(4-chlorophenyl)-3-methyl-1-phenyl-1,4-dihydropyrano[2,3-c]pyrazole-5-carbonitrile (4b)

White powder, m.p. 172°C–173°C (Rep. 174°C–176°C) (Vafajoo et al., 2015); FTIR (KBr) υ_max_/cm^−1^: 3,445, 3,322, 2,923, 2197,1663, 1,562; ^1^H NMR (300 MHz, DMSO-*d*_*6*_): *δ* = 1.80 (s, 3H, CH_3_), 4.73 (s, 1H, benzilic), 7.28-7.35 (m, 5H, aromatic), 7.40-7.43 (m, 2H, aromatic), 7.50 (t, 2H, *J =* 7.5 Hz, aromatic), 7.79 (d, 2H, *J* = 7.8* *Hz, aromatic) ppm (Supplementary Figure S2).

#### 4.1.3 6-amino-3-methyl-4-(4-nitrophenyl)-1-phenyl-1,4-dihydropyrano[2,3-c]pyrazole-5-carbonitrile (4e)

White powder, m.p. 192°C–193°C (Rep. 190°C–195°C) (Vafajoo et al., 2015); FTIR (KBr) υ_max_/cm^−1^: 3,446, 3,332, 2,913, 2,193, 1,668, 1,552, 1,351; ^1^H NMR (300 MHz, DMSO-*d*_*6*_): *δ* = 1.80 (s, 3H, CH_3_), 4.93 (s, 1H, benzilic), 7.34-7.40 (m, 3H, aromatic), 7.51-7.60 (m, 4H, aromatic), 7.80 (d, 2H, *J =* 6.6 Hz, aromatic), 8.24 (d, 2H, *J =* 6.9 Hz, aromatic)ppm (Supplementary Figure S3).

#### 4.1.4 6-amino-3-methyl-1-phenyl-4-(3,4,5-trimethoxyphenyl)-1,4-dihydropyrano[2,3-c]pyrazole-5-carbonitrile (4g)

White powder, m.p. 202°C–204°C (Rep. 200°C–203°C) (Jasim et al., 2022); FTIR (KBr) υ_max_/cm^−1^: 3,445, 3,342, 2,921, 2192,1661, 1,530, 1,210; ^1^H NMR (300 MHz, DMSO-*d*_*6*_): *δ* = 1.87 (s, 3H, CH_3_), 3.35 (s, 3H, OMe), 3.66 (s, 6H, OMe), 4.67 (s, 1H, benzilic), 6.65 (br, 2H, NH_2_), 7.23-7.34 (m, 3H, aromatic), 7.46-7.51 (m, 2H, aromatic), 7.80 (d, 2H, *J = 7.80* Hz, aromatic) ppm (Supplementary Figure S4).

#### 4.1.5 6-amino-4-(3-ethoxy-4-hydroxyphenyl)-3-methyl-1-phenyl-1,4-dihydropyrano[2,3-c]pyrazole-5-carbonitrile (4j)

White powder, m.p. 172°C–173°C (Rep. 169°C–171°C) (Vafajoo et al., 2015); FTIR (KBr) υ_max_/cm^−1^: 3,445, 3,342, 2,921, 2192,1661, 1,530, 1,210; ^1^H NMR (400 MHz, DMSO-*d*_*6*_): *δ* = 1.31 (t, 3H, *J* = 8 Hz, CH_3_), 1.82 (s, 3H, CH_3_), 3.98 (q, 2H, *J = 8* Hz, OCH_2_), 4.57 (s, 1H, benzilic), 6.62 (d.d, 1H, = 8 Hz, aromatic), 6.74-6.80 (m, 2H), 7.15 (br, 2H, NH_2_), 7.32 (t.d, 1H, *J = 4* Hz, aromatic), 7.49 (t, 2H*, J = 12* Hz, aromatic), 7.79 (d.d, 2H, *J* = *7.80* Hz, aromatic) ppm (Supplementary Figure S5).

#### 4.1.6 6-amino-3-methyl-4-(naphthalen-1-yl)-1-phenyl-1,4-dihydropyrano[2,3-c]pyrazole-5-carbonitril (4k)

White powder, m.p.240°C–242°C (Rep. 244°C–245°C) (Zolfigol et al., 2016); FTIR (KBr) υ_max_/cm^−1^: 3,447, 3,392, 2,911, 2,196, 1,662, 1,550; ^1^H NMR (300 MHz, DMSO-*d*_*6*_): *δ* = 2.30 (s, 3H, CH_3_), 5.61 (s, 1H, benzilic), 7.20-7.24 (m, 2H, aromatic), 7.40-7.55 (m, 5H, aromatic), 7.70-8.01 (m, 7H, aromatic) ppm (Supplementary Figure S6).

#### 4.1.7 6-amino-3-methyl-1-phenyl-4-(thiophen-2-yl)-1,4-dihydropyrano[2,3-c]pyrazole-5-carbonitrile (4o)

White powder, m.p.168°C–170°C (Rep. 167°C–168°C) (Zolfigol et al., 2016); FTIR (KBr) υ_max_/cm^−1^: 3,447, 3,392, 2,911, 2,196, 1,662, 1,550; ^1^HNMR(400 MHz, DMSO-*d*_*6*_): *δ* = 1.93 (s, 3H, CH_3_), 5.10 (s, 1H, benzilic), 6.98-7.00 (m, 1H,aromatic), 7.08 (d.d, 1H, *J = 8* Hz, aromatic), 7.32-7.35 (m, 2H, aromatic), 7.44 (d, 2H, *J = 4* Hz), 7.50 (t, 1H, *J = 8* Hz, aromatic), 7.79 (d.d, 2H*, J = 8* Hz, aromatic) ppm (Supplementary Figure S7).

#### 4.1.8 4,4'-((4-hydroxy-3-methoxyphenyl)methylene)bis(3-methyl-1-phenyl-1H-pyrazol-5-ol) (5i)

White powder, m.p.206°C–208°C (Rep. 206°C–207°C) (Zolfigol et al., 2015); FTIR (KBr) υ_max_/cm^−1^: 3,451, 3,286, 2,941, 1,617; ^1^H NMR (300 MHz, DMSO-*d*_*6*_): *δ* = 2.31 (s, 6H, CH_3_),3.14 (s, 3H, OCH_3_), 4.85 (s, 1H, benzilic), 6.69 (br, 2H, aromatic), 6.86 (s, 1H, aromatic), 7.24 (t, 4H, *J = 7.2* Hz, aromatic), 7.71 (d, 4H, *J = 7.5* Hz, aromatic), 8.76(br, 1H, OH), 14.02 (br, 2H, OH) ppm (Supplementary Figure S8).

#### 4.1.9 4,4'-(naphthalen-1-ylmethylene)bis(3-methyl-1-phenyl-1H-pyrazol-5-ol) (5k)

White powder, m.p.212°C–214°C (Rep. 210°C–212°C) (Jahanshahi and Akhlaghinia, 2017); FTIR (KBr) υ_max_/cm^-1^3420, 3,176, 2,940, 1619,1105; ^1^H NMR (400 MHz, DMSO-*d*_*6*_): *δ* = 2.30 (s, 6H, CH_3_), 5.62 (s, 1H, benzilic), 7.21-7.24 (m, 2H, aromatic), 7.41-7.55 (m, 7H, aromatic), 7.65-7.73 (m, 5H, aromatic), 7.93-8.02 (m, 2H, aromatic), 13.18 (br, 2H, OH) ppm (Supplementary Figure S9).

#### 4.1.10 4,4'-((2-methoxyphenyl)methylene)bis(3-methyl-1-phenyl-1H-pyrazol-5-ol) (5o)

White powder, m.p.214°C–215°C (Rep. 212°C–213°C) (Jahanshahi and Akhlaghinia, 2017); FTIR (KBr) υ_max_/cm^−1^: 3,431, 3,176, 2,946, 1,621, 1,115 ^1^H NMR (400 MHz, DMSO-*d*_*6*_): *δ* = 2.30 (s, 6H, CH_3_), 3.38 (s, 3H, OCH_3_), 5.21 (s, 1H, benzilic), 6.87-6.97 (m, 2H, aromatic), 7.15-7.28 (m, 3H, aromatic), 7.45 (t, 4H, *J = 7.8* Hz, aromatic), 7.63 (d, 1H, *J = 6.6* Hz, aromatic), 7.72 (d, 4H*, J = 7.8* Hz, aromatic), 14.41 (br, 2H, OH) ppm (Supplementary Figure S10).

#### 4.1.11 4,4′,4″,4‴-(((hexane-1,6-diylbis(oxy))bis(3-methoxy-4,1-phenylene))bis(methanetriyl)) tetrakis(3-methyl-1-phenyl-1H-pyrazol-5-ol) (6a)

White powder, m.p.196°C–198°C; FTIR (KBr) υ_max_/cm^−1^: 3,418, 3,228, 2,967, 1,609, 1,535; ^1^H NMR (400 MHz, DMSO-d_6_): *δ* = 1.43 (br, 2H, CH_2,_ bridge), 1.68 (br, 2H, CH_2,_ bridge), 2.31 (s, 6H, CH_3_), 3.64 (s, 3H, OCH_3_), 3.88 (m, 2H, OCH_2,_ bridge), 4.87 (s, 1H, benzilic), 6.79-6.78 (m, 3H, aromatic), 7.24 (t, 2H, J = 7.2 Hz,aromatic), 7.44 (t, 4H, J = 8 Hz, aromatic), 7.70 (d, 4H, J = 7.6 Hz, aromatic), 14.03 (s, 1H, OH) ppm. ^13^C NMR (100 MHz, DMSO-d_6_): *δ* = 25.77, 29.24, 33.35, 56.04, 56.50, 68.64, 112.38, 113.49, 119.84, 121.06, 126.04, 129.39, 135.42, 146.62, 147.07, and 149.09 ppm (Supplementary Figure S11).

#### 4.1.12 4,4'-((4-((6-(4-((5-hydroxy-1-phenyl-1H-pyrazol-4-yl)(5-hydroxy-3-methyl-1-phenyl-1H-pyrazol-4-yl)methyl)phenoxy)hexyl)oxy)phenyl)methylene)bis(3-methyl-1-phenyl-1H-pyrazol-5-ol) (6b)

White powder, m.p.192°C–194°C; FTIR (KBr) υ_max_/cm^−1^: 3,423, 3,211, 2,973, 1,611, 1,528; ^1^H NMR (400 MHz, DMSO-d_6_): *δ* = 1.43 (br, 2H, CH_2,_ bridge), 1.67 (br, 2H, CH_2,_ bridge), 2.30 (s, 6H, CH_3_), 3.89 (t, 2H, J = 6.4 Hz, OCH_2,_ bridge), 4.88 (s, 1H, benzilic), 6.81 (d, 2H, J = 8.8 Hz, aromatic), 7.13 (d, 2H, J = 8.4 Hz, aromatic),7.24 (t, 2H, J = 7.2 Hz,aromatic), 7.43 (t, 4H, J = 7.6 Hz, aromatic), 7.70 (d, 4H, J = 7.6 Hz, aromatic), 13.94 (s, 1H, OH) ppm. ^13^C NMR (100 MHz, DMSO-d_6_): *δ* = 25.81, 29.16, 32.80, 56.50, 67.71, 114.48, 115.36, 120.96, 126.01, 128.60, 129.38, 132.28, 134.36, 146.64, and 157.39 ppm (Supplementary Figure S12).

#### 4.1.13 4,4′,4″,4‴-(((butane-1,4-diylbis(oxy))bis(3-methoxy-4,1-phenylene)) bis(methanetriyl)) tetrakis(3-methyl-1-phenyl-1H-pyrazol-5-ol) (6c)

White powder, m.p.140°C–142°C; FTIR (KBr) υ_max_/cm^−1^: 3,423, 3,236, 2,951, 1618,1103; ^1^H NMR (400 MHz, DMSO-d_6_): *δ* = 1.82 (br, 2H, CH_2,_ bridge), 2.31 (s, 6H, CH_3_), 3.83 (s, 3H,OCH_3_), 4.17 (br, 2H, OCH_2,_ bridge), 4.88 (s, 1H, benzilic), 6.78-6.88 (m, 3H, aromatic), 7.24 (t, 2H, J = 7.2 Hz, aromatic), 7.44 (t, 4H, J = 8 Hz, aromatic), 7.70 (d, 4H, J = 8 Hz, aromatic), 14.03 (s, 1H, OH) ppm. ^13^C NMR (100 MHz, DMSO-d_6_): *δ* = 25.72, 33.36, 55.98, 56.51, 38.45, 112.44, 113.51, 119.81, 121.06, 126.05, 129.39, 135.47, 146.65, 149.67, and 154.00 ppm (Supplementary Figure S13).

#### 4.1.14 4,4′,4″,4‴-(((hexane-1,6-diylbis(oxy))bis(2,1-phenylene))bis(methanetriyl))tetrakis(3-methyl-1-phenyl-1H-pyrazol-5-ol) (6d)

White powder, m.p.169°C–172°C; FTIR (KBr) υ_max_/cm^−1^: 3,422, 3,225, 2,963, 1,615, 1,541, ^1^H NMR (400 MHz, DMSO-d_6_): *δ* = 1.52 (br, 2H, CH_2,_ bridge), 1.79 (br, 2H, CH_2,_ bridge), 2.29 (s, 6H, CH_3_), 4.01 (br, 2H, OCH_2,_ bridge), 5.25 (s, 1H, benzilic), 6.86 (t, 1H, J = 7.2 Hz,aromatic), 6.92 (d, 1H, J = 8.4 Hz, aromatic), 7.14 (t, 1H, J = 7.6 Hz,aromatic), 7.23 (t, 2H, J = 7.2 Hz, aromatic), 7.43 (t, 4H, J = 8 Hz, aromatic), 7.67-7.72 (m, 5H, aromatic), 14.23 (s, 1H, OH) ppm. ^13^C NMR (100 MHz, DMSO-d_6_): *δ* = 25.91, 27.61, 29.27, 56.50, 68.05, 11.86, 120.29, 121.07, 126.08, 127.76, 128.05, 129.37, 131.17, 146.43, and 155.41 ppm (Supplementary Figure S14).

#### 4.1.15 4,4′,4″,4‴-(1,4-phenylenebis(methanetriyl))tetrakis(3-methyl-1-phenyl-1H-pyrazol-5-ol) (6e)

White powder, m.p.194°C–196°C (Rep. 193°C–196°C) (Hasaninejad et al., 2011); FTIR (KBr) υ_max_/cm^−1^: 3,419, 3,229, 2,981, 1628,1105; ^1^H NMR (400 MHz, DMSO-d_6_): *δ* = 2.30 (s, 6H, CH_3_), 4.89 (s, 1H, benzilic), 7.18 (s, 2H, aromatic), 7.24 (t, 2H, J = 6.8 Hz, aromatic), 7.42 (t, 4H, J = 7.6 Hz, aromatic), 7.68 (d, 4H, J = 8 Hz, aromatic), 14.07 (br, 2H, OH) ppm. ^13^C NMR (100 MHz, DMSO-d_6_): *δ* = 33.23, 66.82, 121.12, 126.16, 127.46, 129.36, 140.44, and 146.68 ppm (Supplementary Figure S15).

## Data Availability

The raw data supporting the conclusion of this article will be made available by the authors, without undue reservation.
