# Supplementary material for: Poly(aniline-co-melamine)@MnFe2O4 nanocatalyst for the synthesis of 4,4′-(arylmethylene) bis (1H-pyrazole-5-ol) derivatives, and 1,4- dihydropyrano[2,3-c]pyrazoles and evaluation of their antioxidant, and anticancer activities
Source: Front Chem. 2022 Oct 28;10:1046120. doi: 10.3389/fchem.2022.1046120 (PMC9649443; doi:10.3389/fchem.2022.1046120)
Supplement: Supplementary file 1 [file DataSheet1.docx]

Supplementary Material


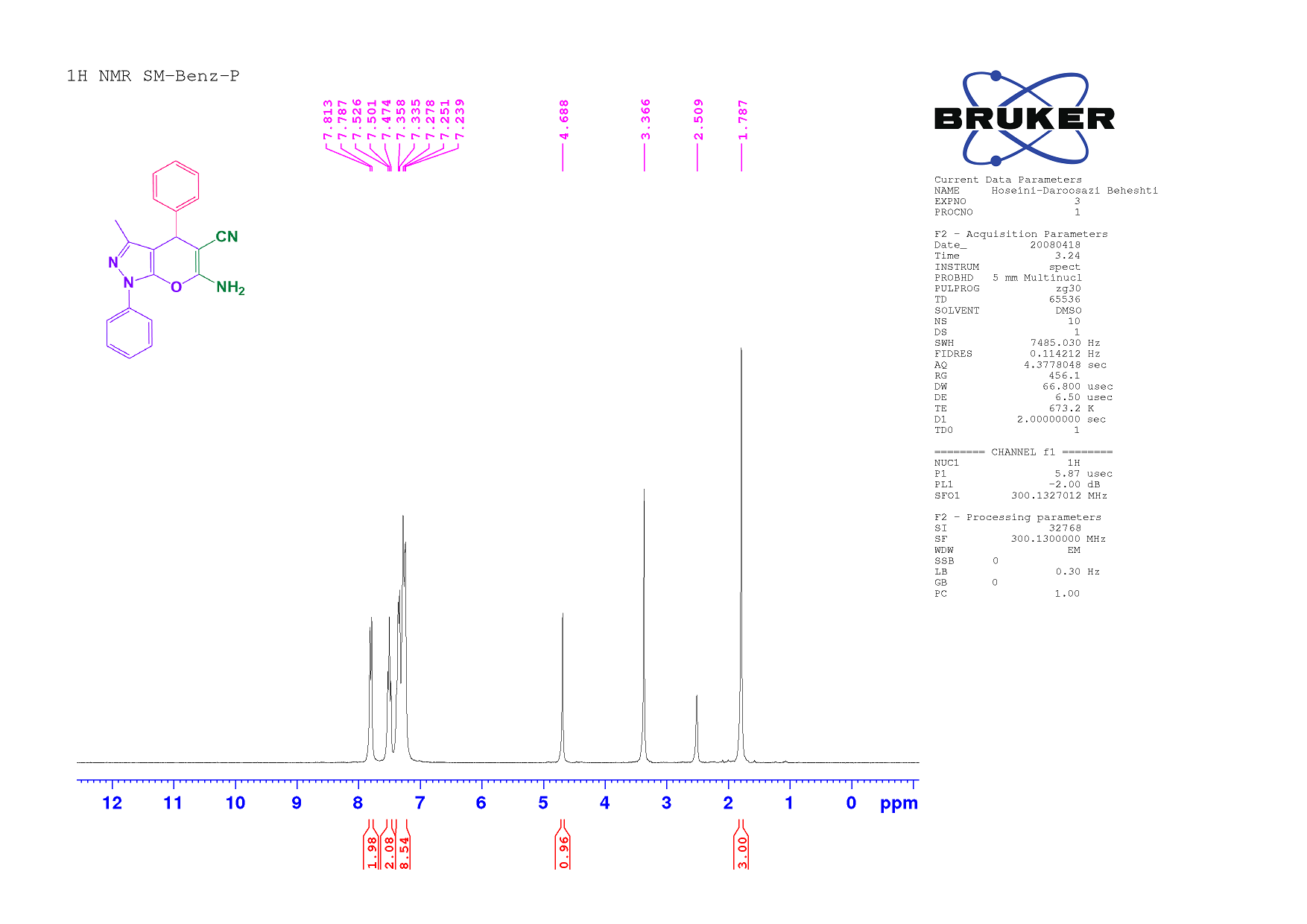


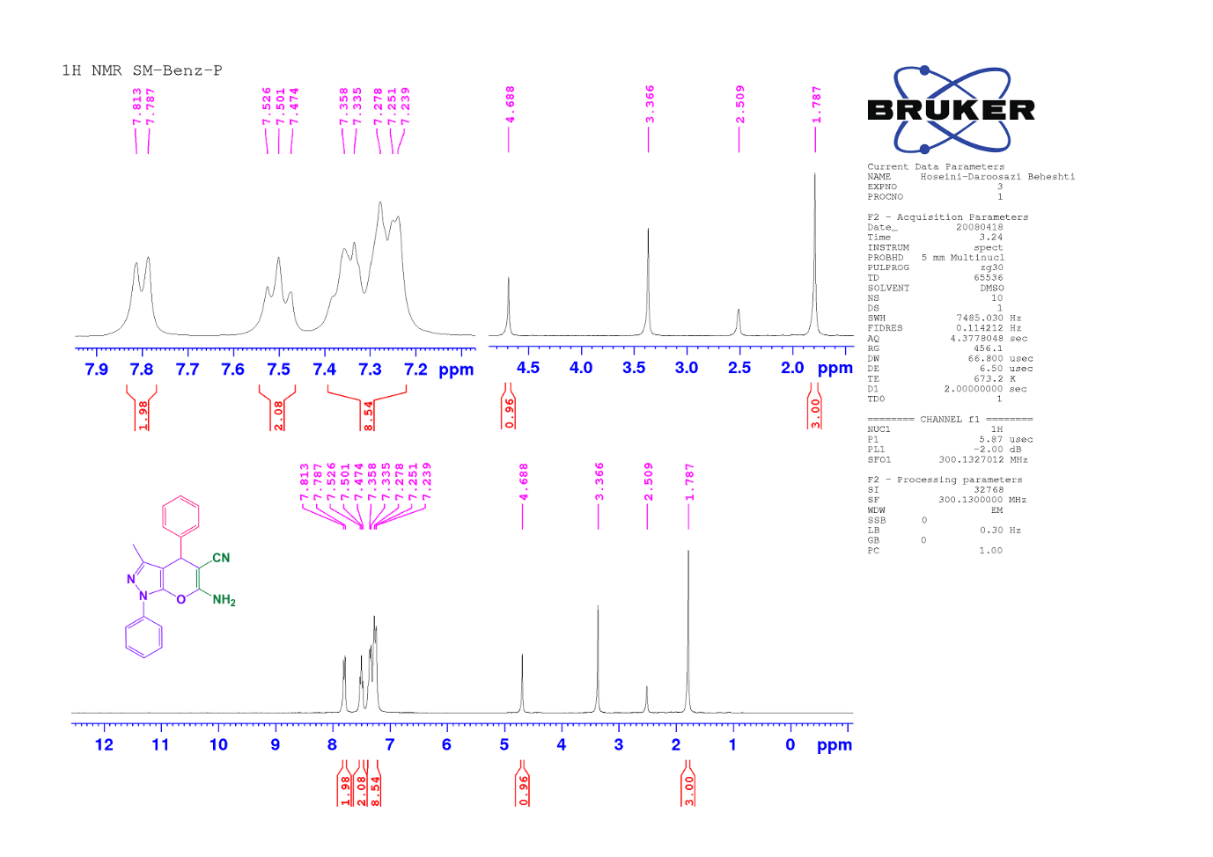


**Figure 1S.** 6-amino-3-methyl-1,4-diphenyl-1,4-dihydropyrano[2,3-c]pyrazole-5-carbonitrile **(4a)**


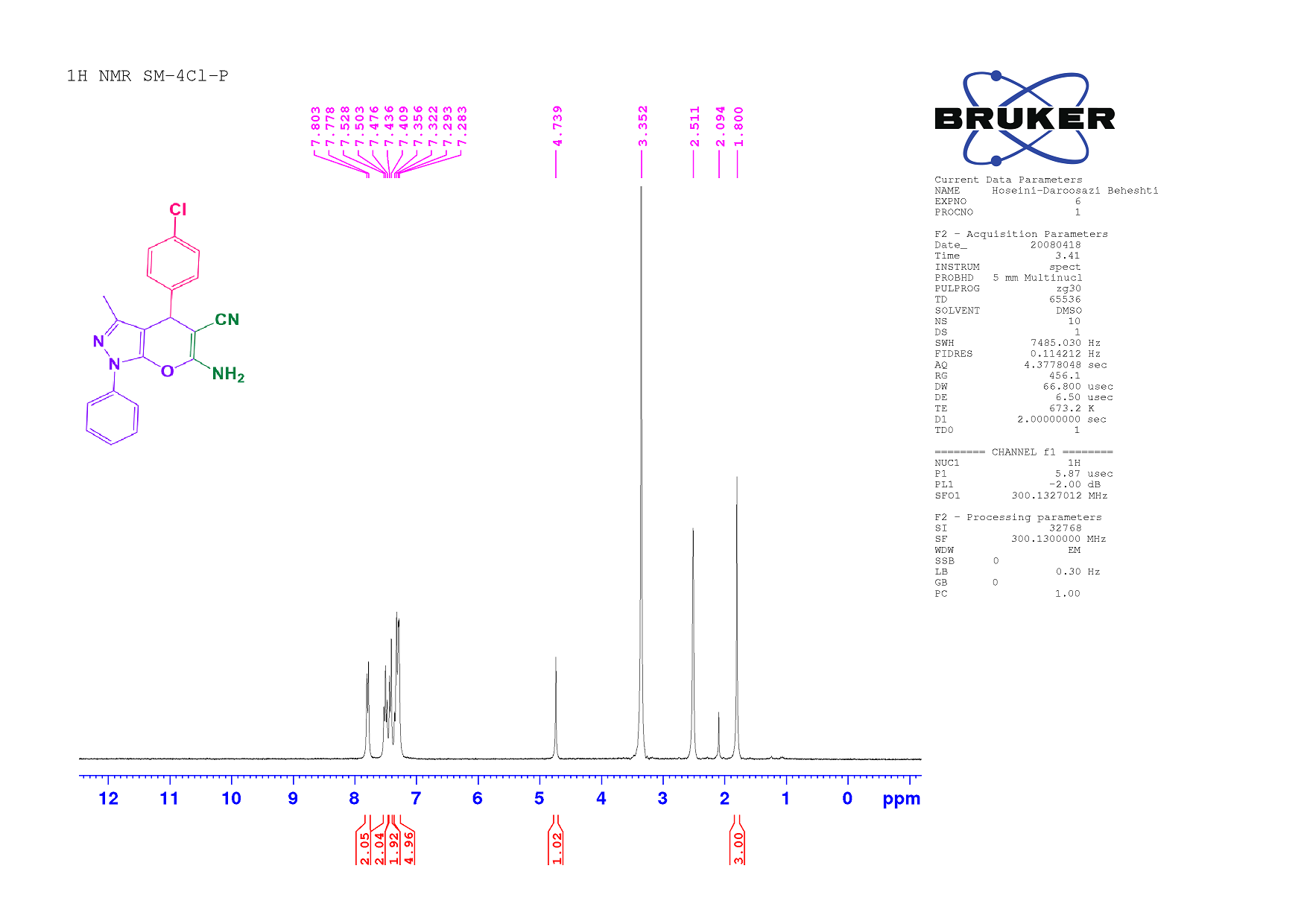


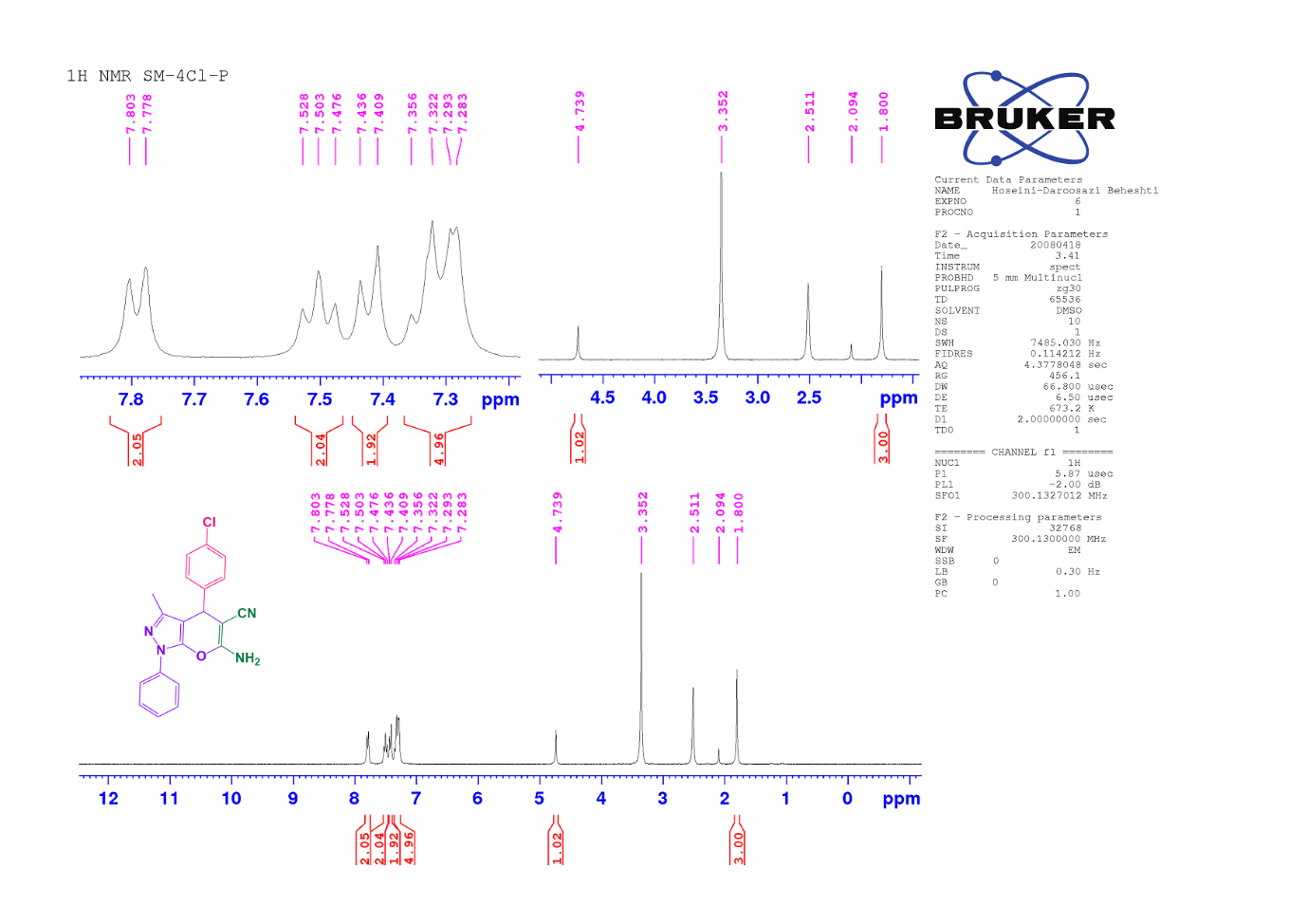


**Figure 2S.** 6-amino-4-(4-chlorophenyl)-3-methyl-1-phenyl-1,4-dihydropyrano[2,3-c]pyrazole-5-carbonitrile **(4b)**


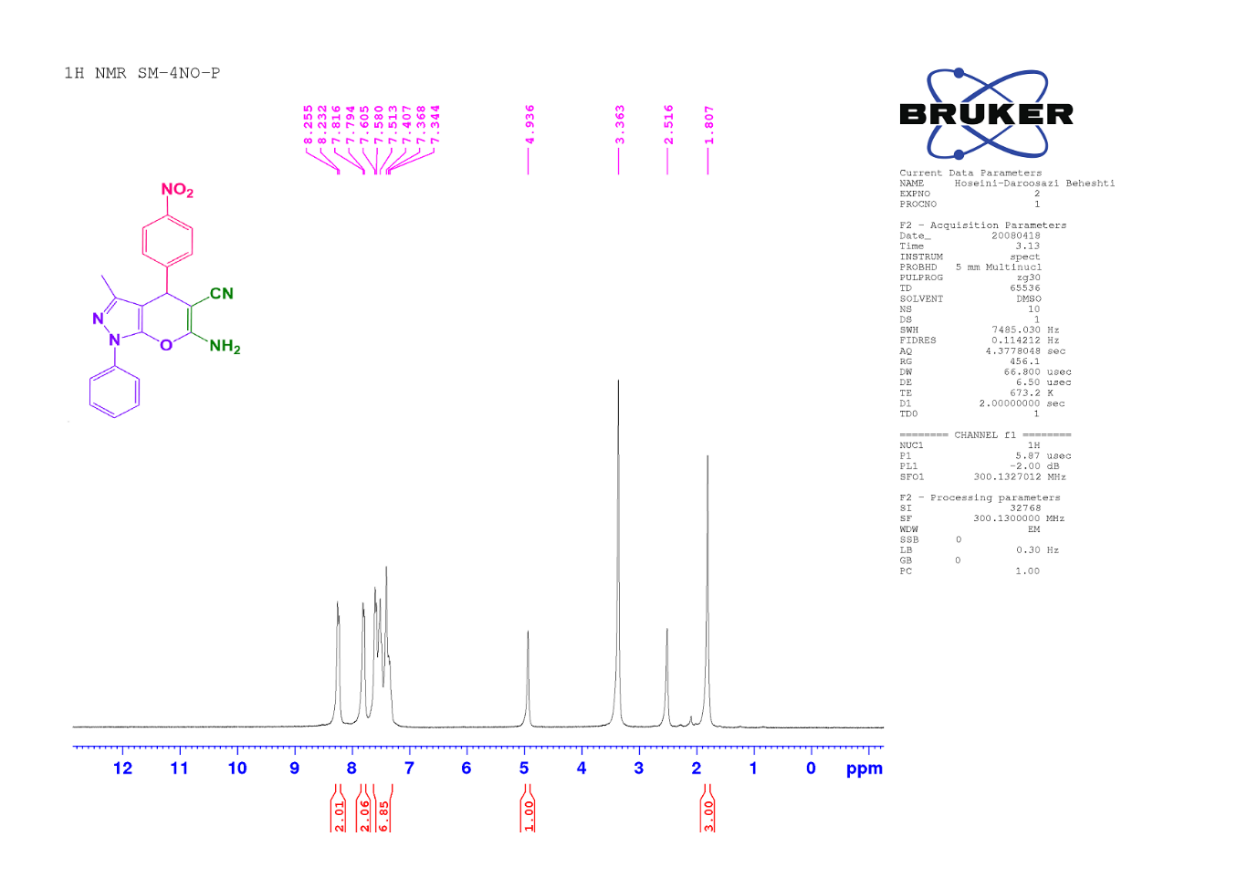


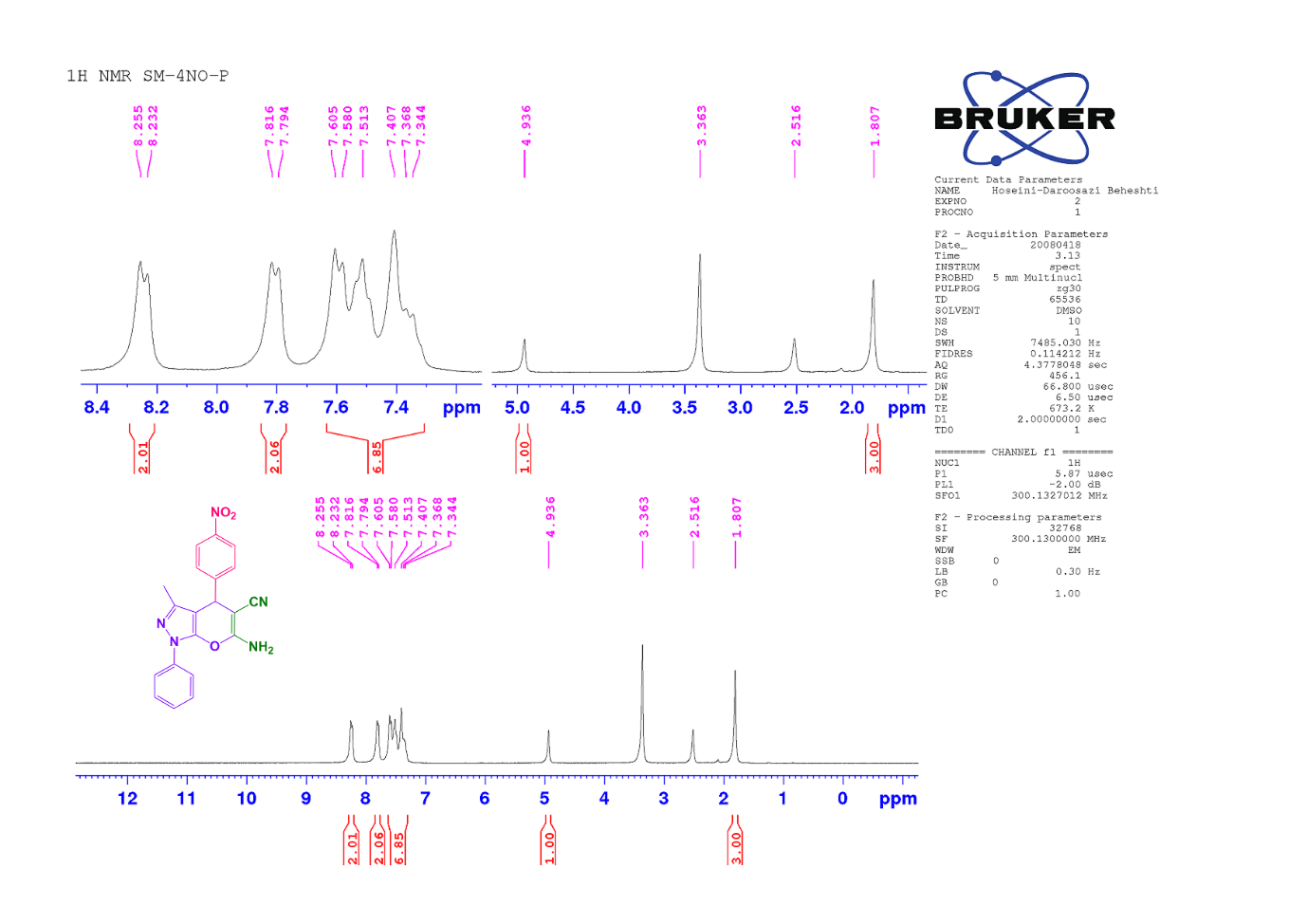


**Figure 3S.** 6-amino-3-methyl-4-(4-nitrophenyl)-1-phenyl-1,4-dihydropyrano[2,3-c]pyrazole-5-carbonitrile **(4e)**


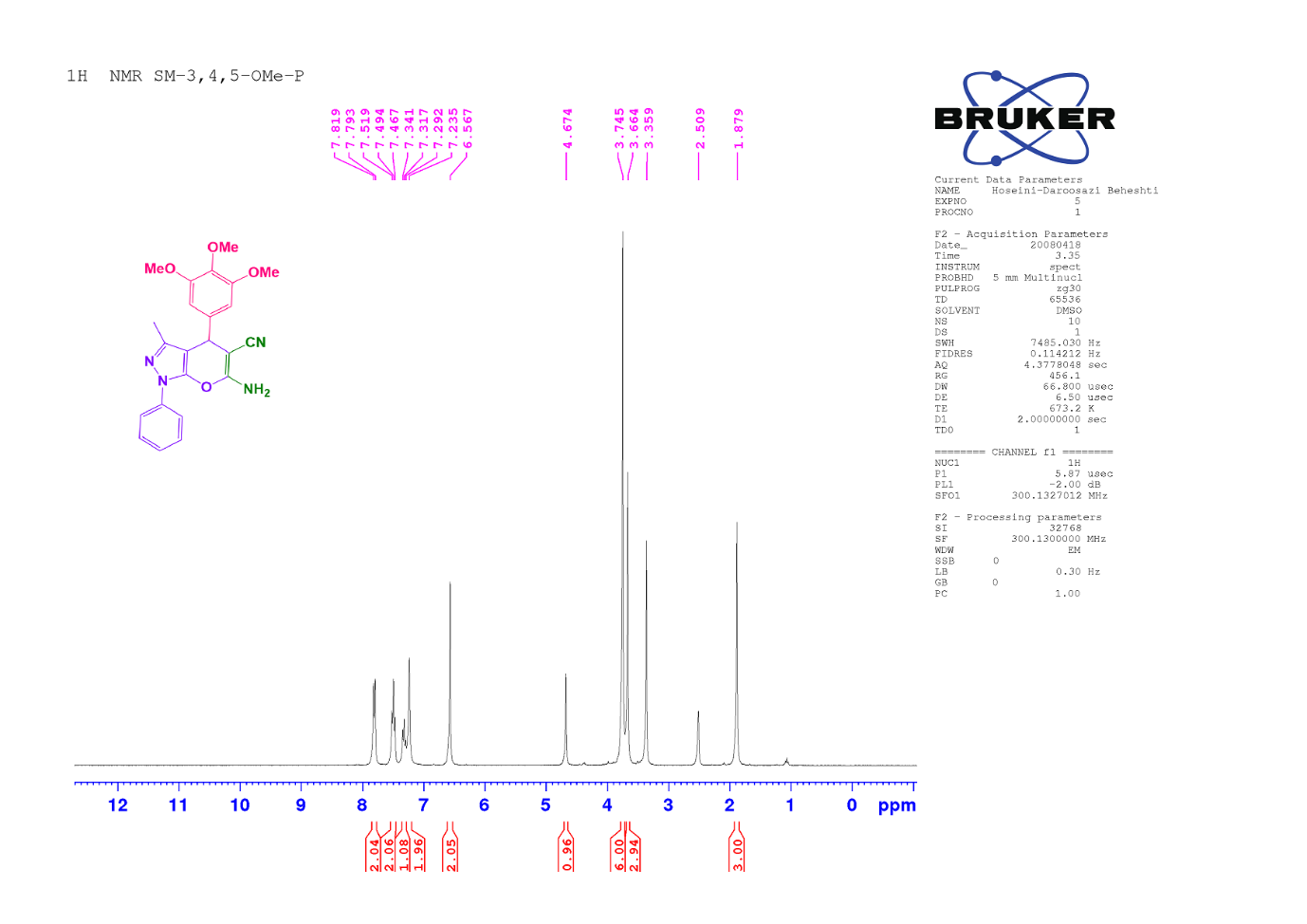


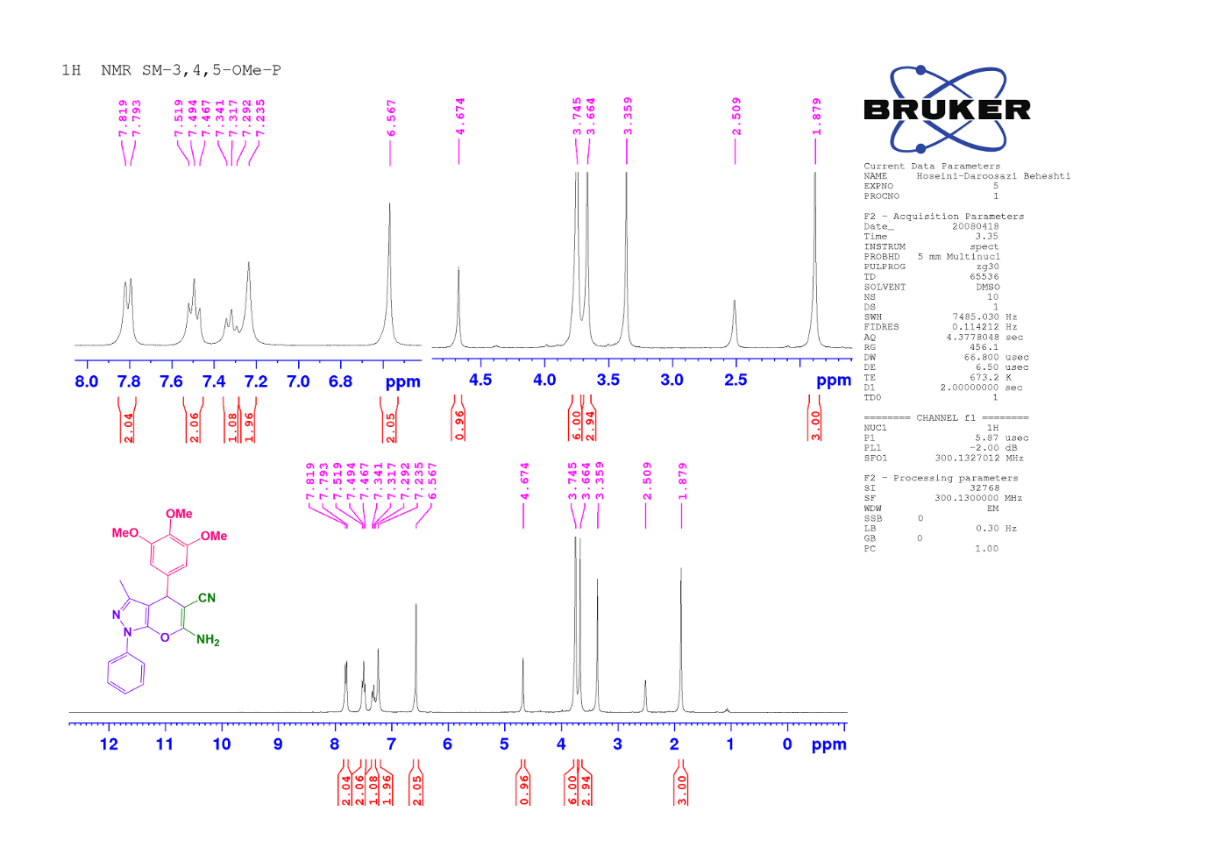


**Figure 4S.** 6-amino-3-methyl-1-phenyl-4-(3,4,5-trimethoxyphenyl)-1,4-dihydropyrano[2,3-c]pyrazole-5-carbonitrile **(4g)**


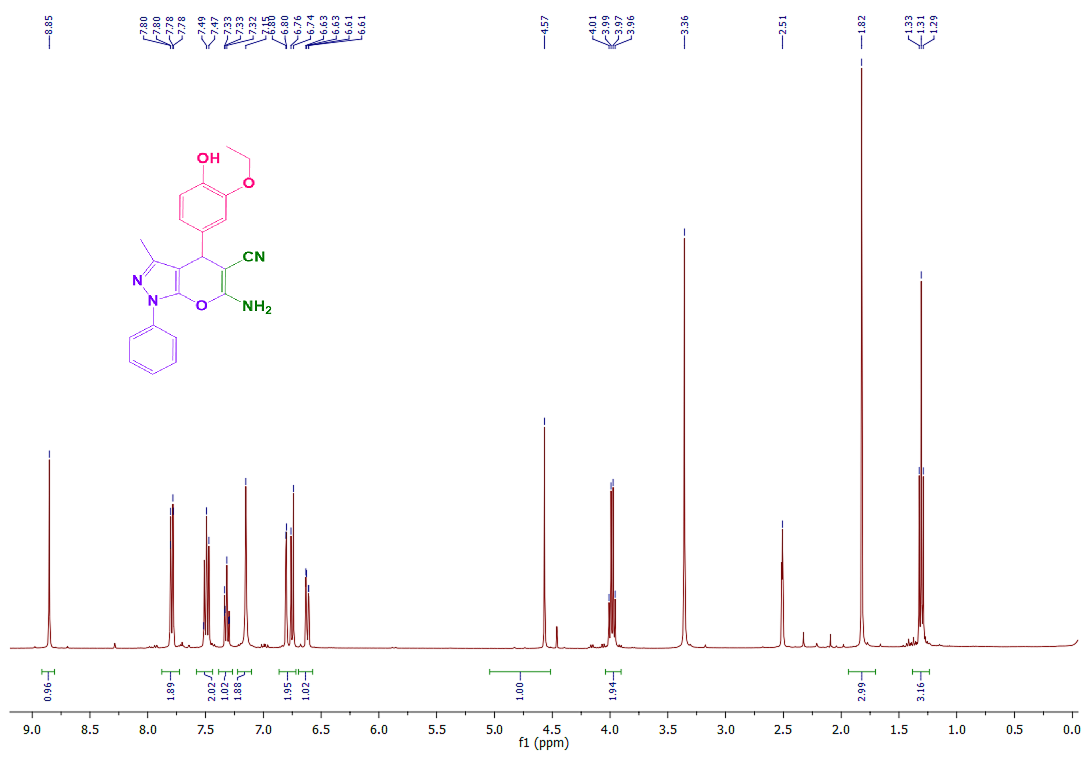


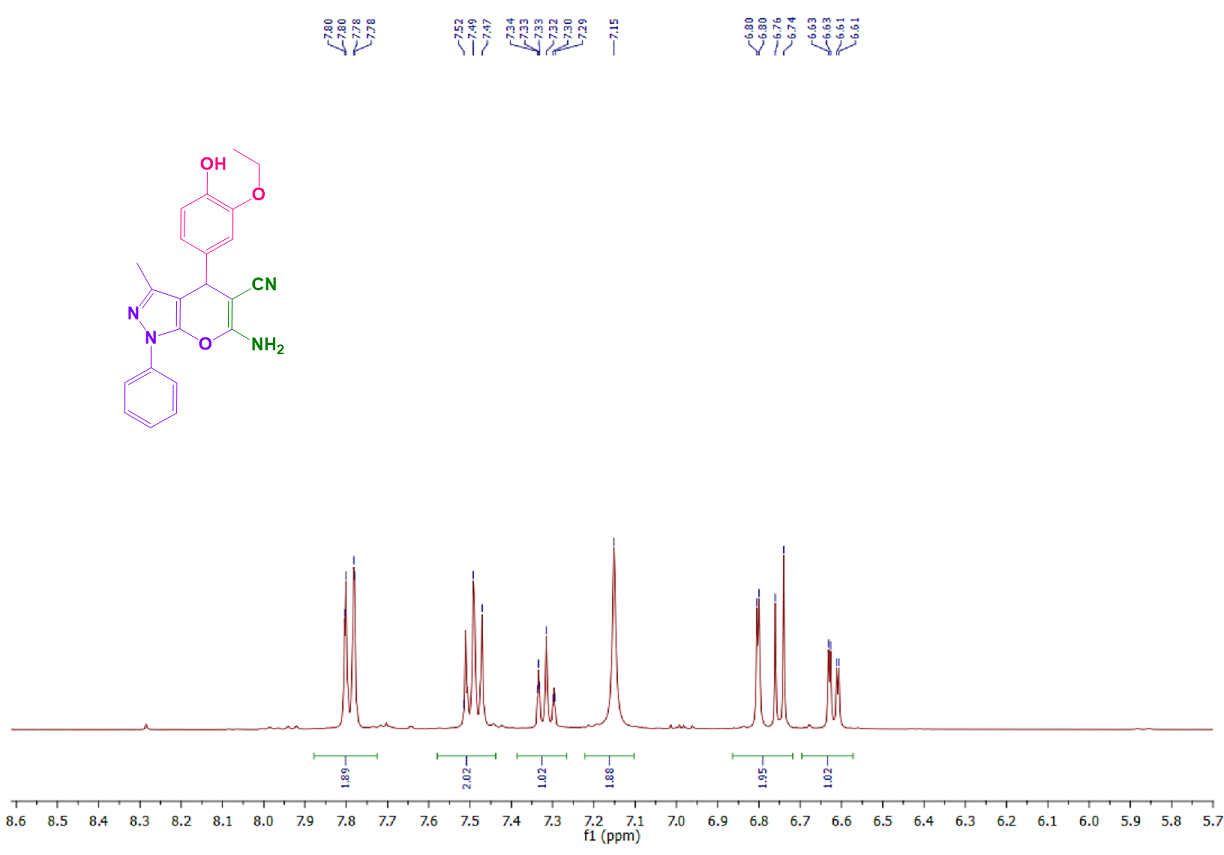


**Figure 5S.** 6-amino-4-(3-ethoxy-4-hydroxyphenyl)-3-methyl-1-phenyl-1,4-dihydropyrano[2,3-c]pyrazole-5-carbonitrile **(4j)**


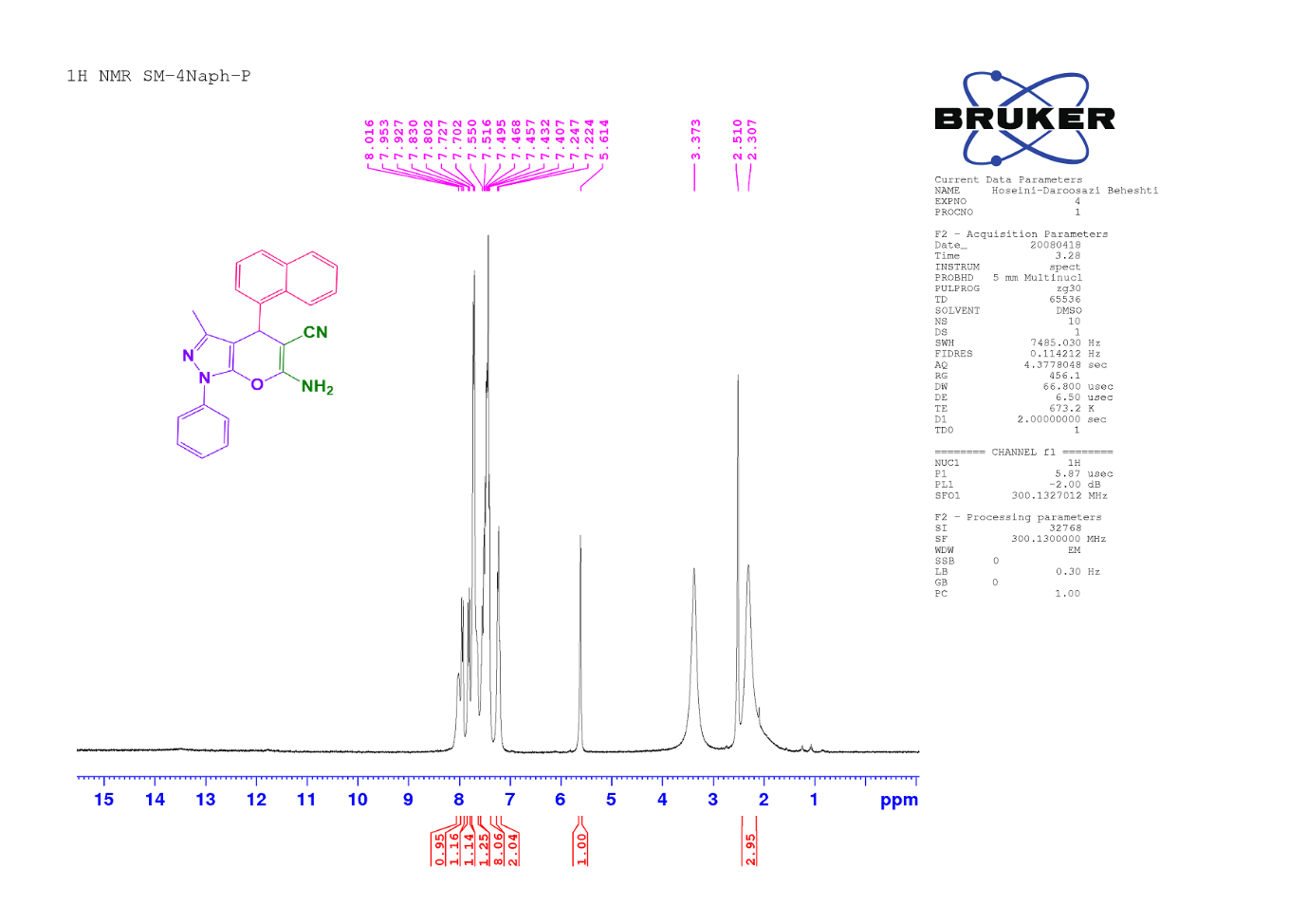


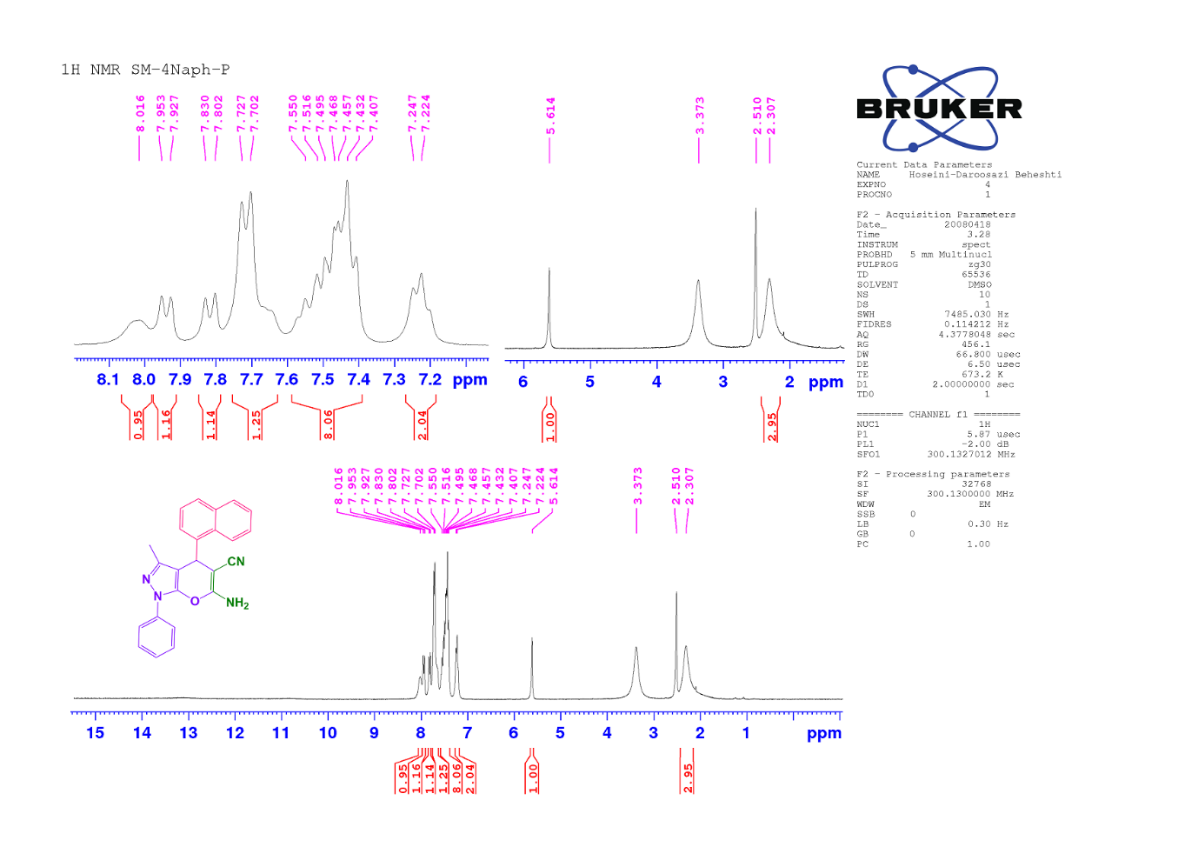


**Figure 6S.** 6-amino-3-methyl-4-(naphthalen-1-yl)-1-phenyl-1,4-dihydropyrano[2,3-c]pyrazole-5-carbonitrile **(4k)**


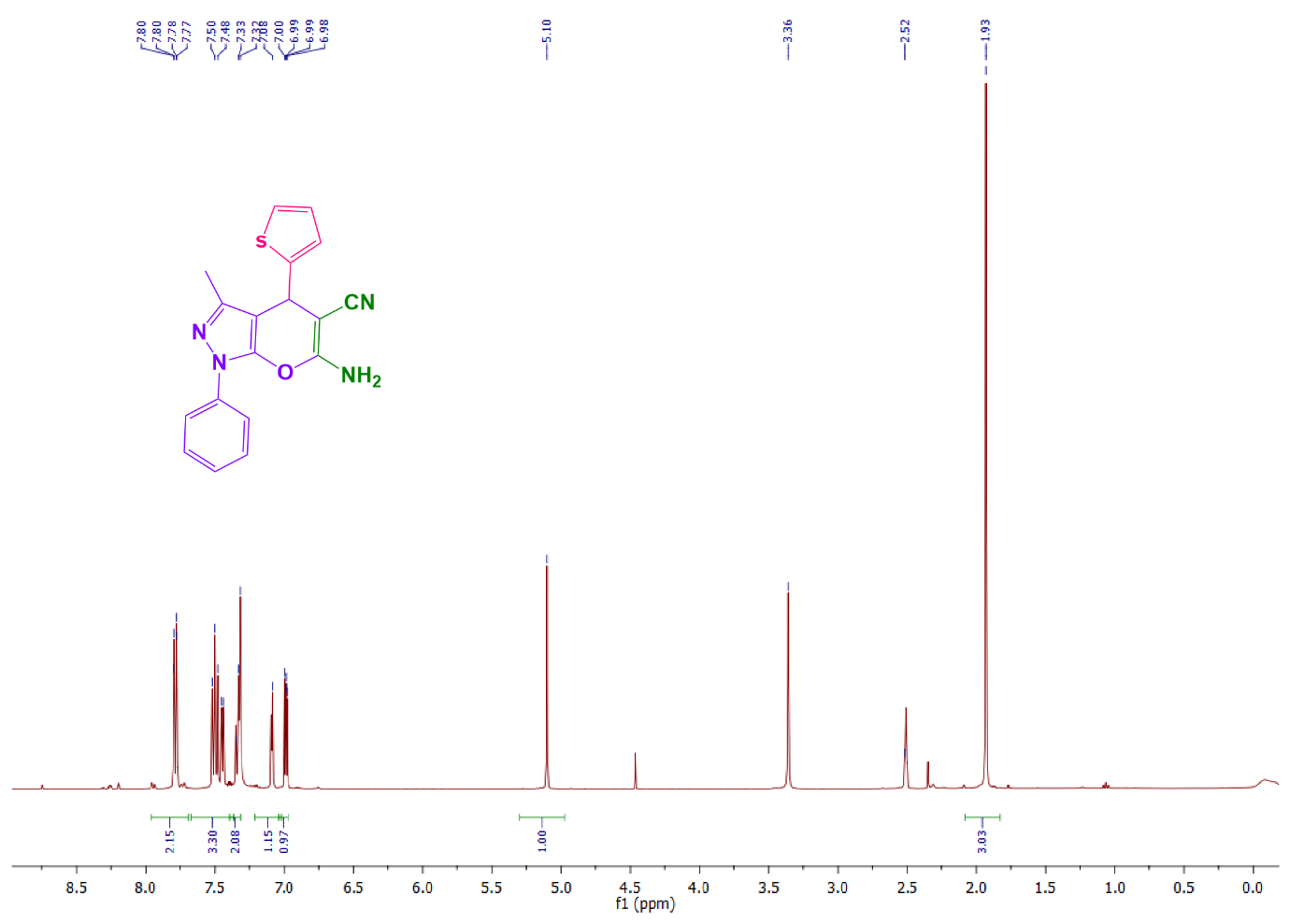


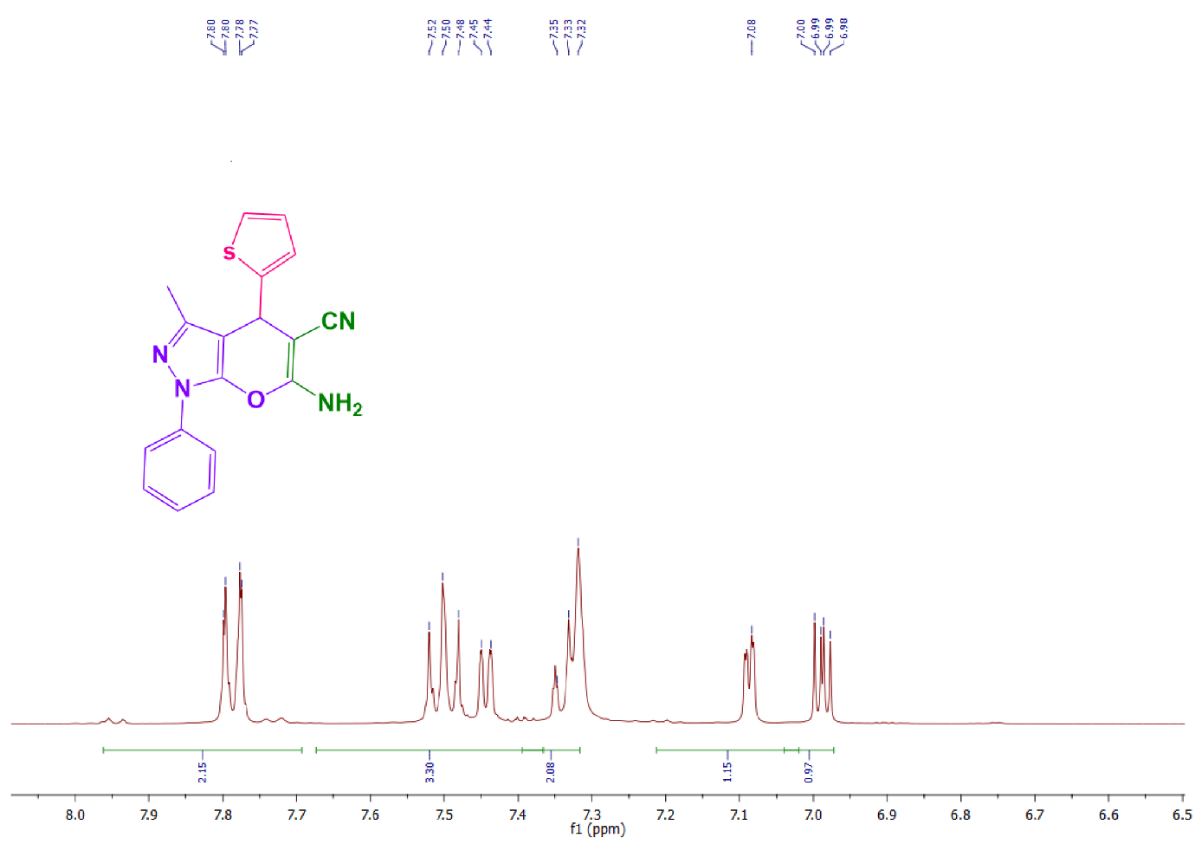


**Figure 7S.** 6-amino-3-methyl-1-phenyl-4-(thiophen-2-yl)-1,4-dihydropyrano[2,3-c]pyrazole-5-carbonitrile **(4o)**


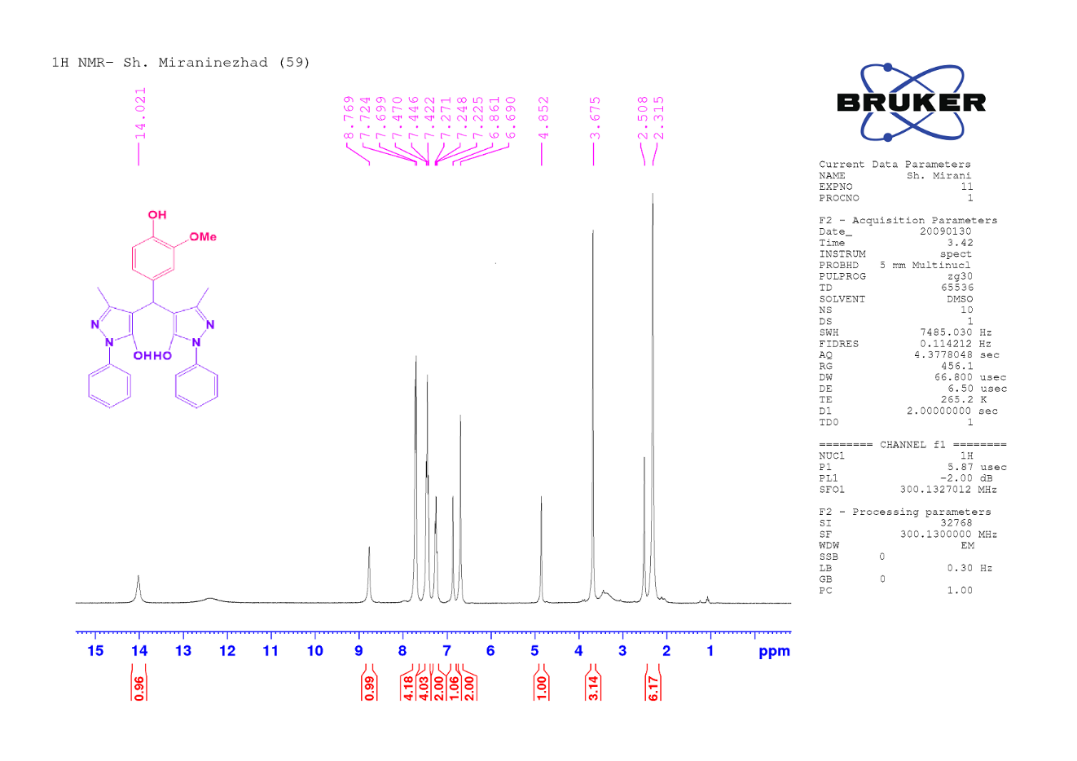


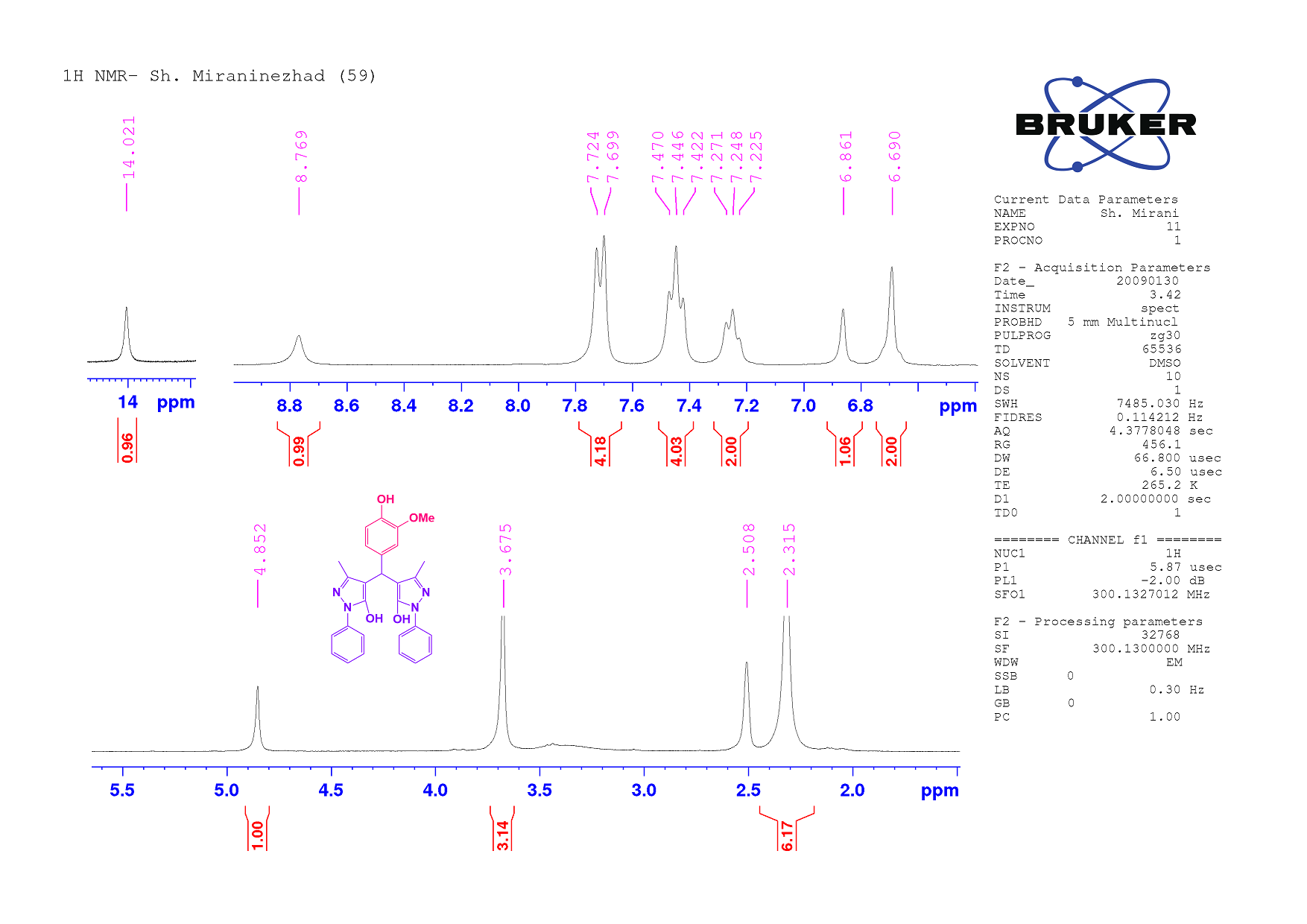


**Figure 8S.** 4,4'-((4-hydroxy-3-methoxyphenyl)methylene)bis(3-methyl-1-phenyl-1H-pyrazol-5-ol) (5i)


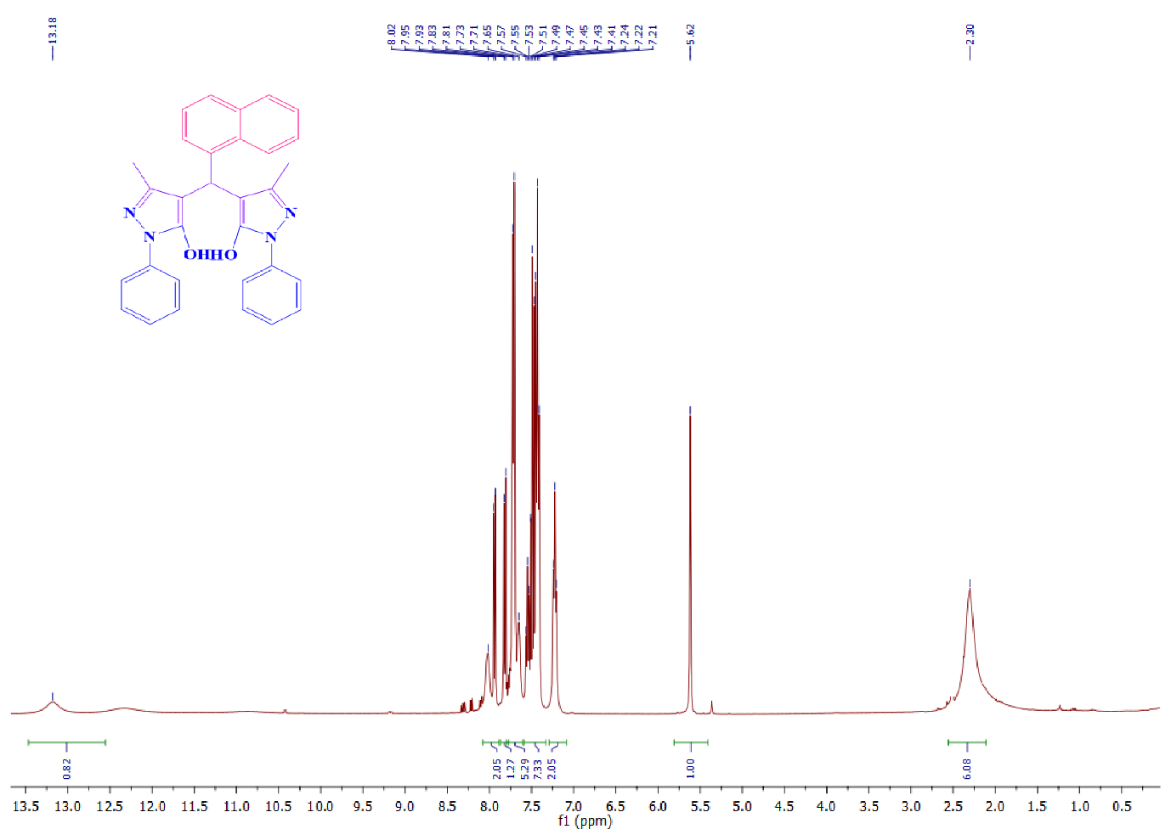


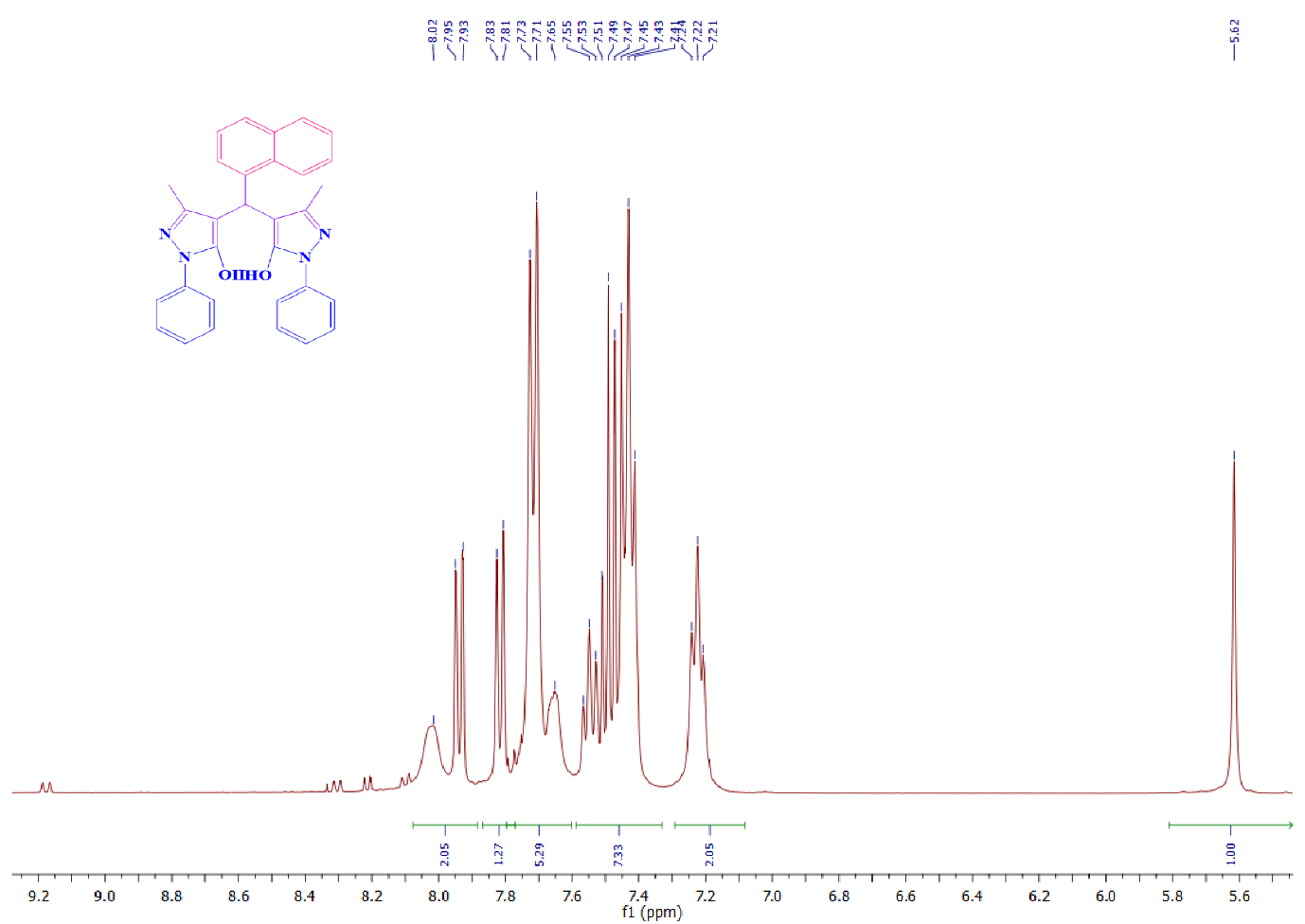


**Figure 9S.** 4,4'-(naphthalen-1-ylmethylene)bis(3-methyl-1-phenyl-1H-pyrazol-5-ol) **(5k)**


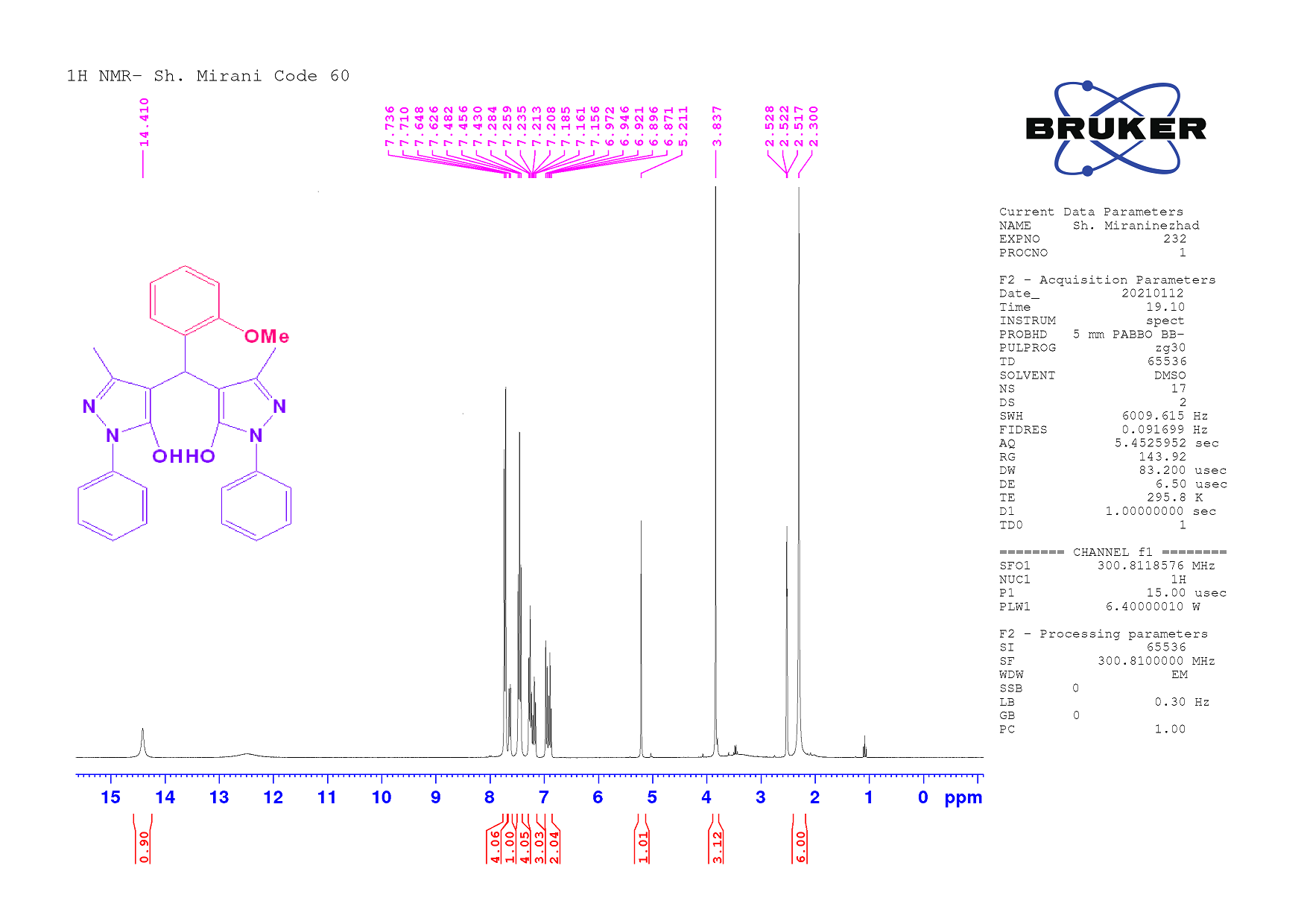


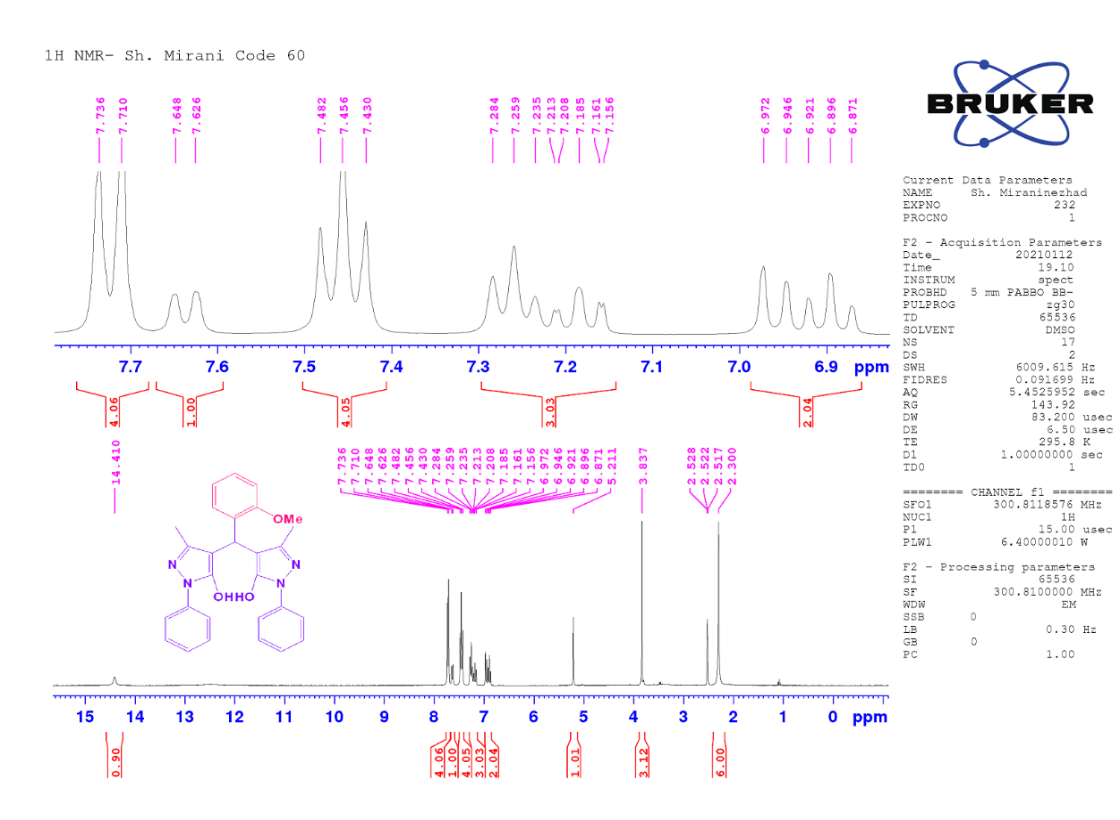


**Figure 10S.** 4,4'-((2-methoxyphenyl)methylene)bis(3-methyl-1-phenyl-1H-pyrazol-5-ol) **(5o)**


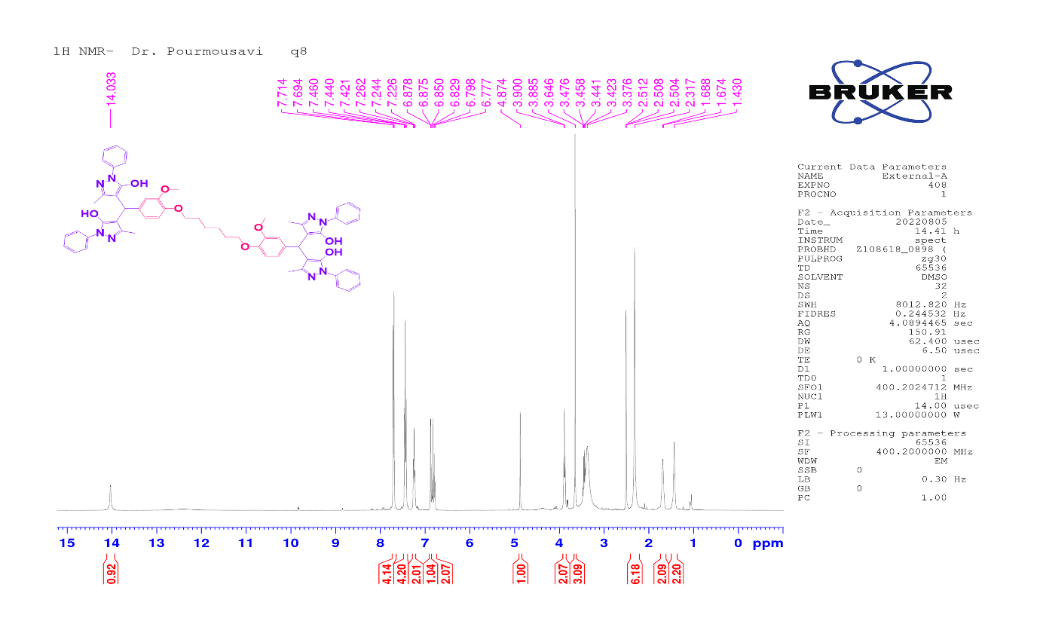


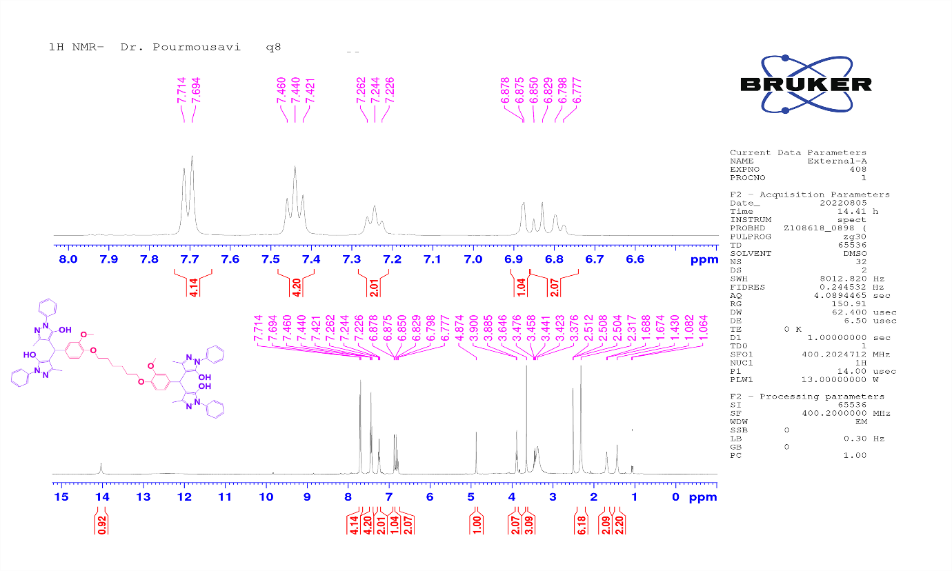


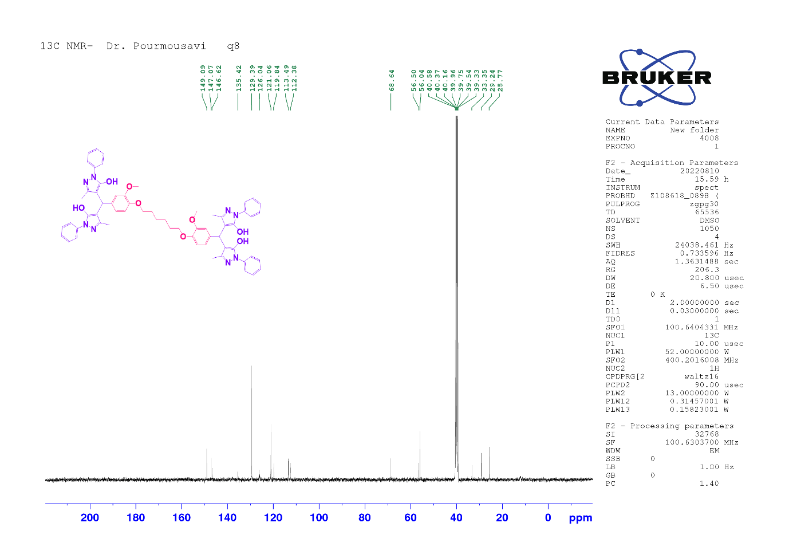


**Figure 11S.** 4,4',4'',4'''-(((hexane-1,6-diylbis(oxy))bis(3-methoxy-4,1-phenylene))bis(methanetriyl)) tetrakis(3-methyl-1-phenyl-1H-pyrazol-5-ol)**(6a)**


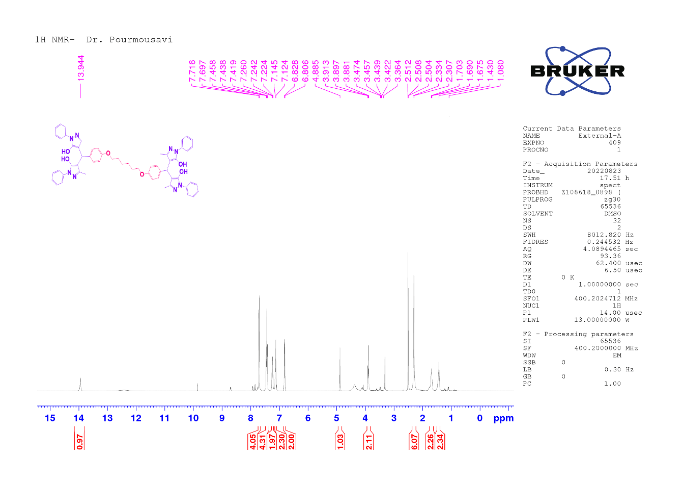


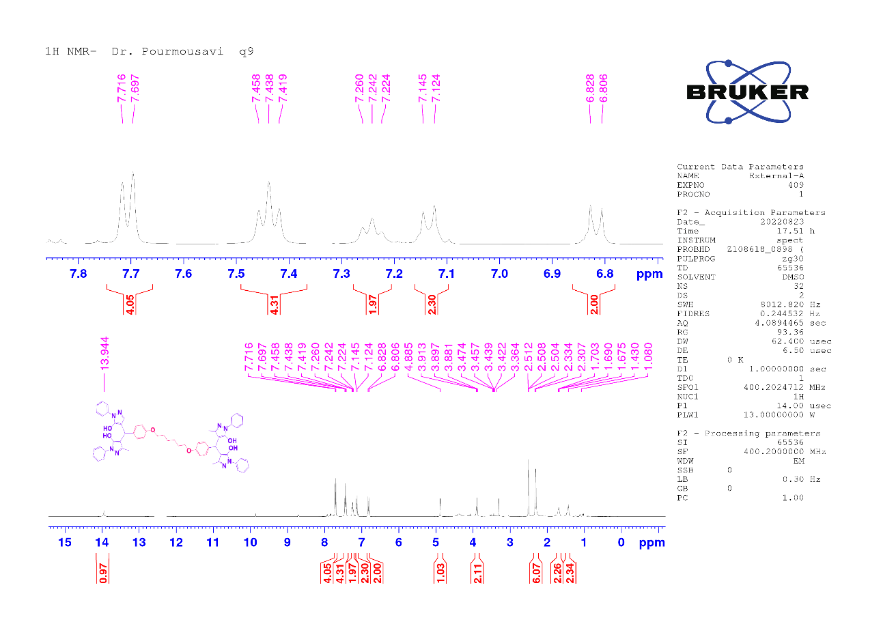


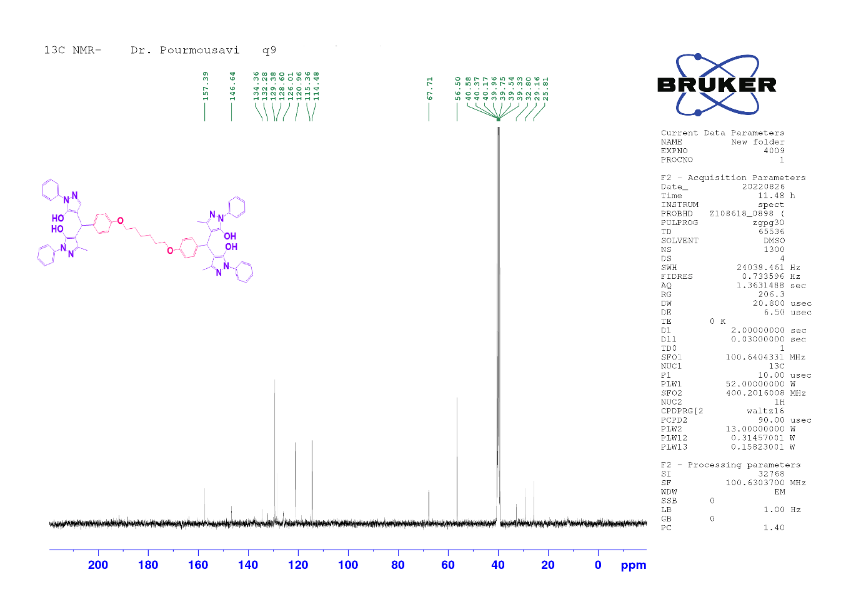


**Figure12S.** 4,4'-((4-((6-(4-((5-hydroxy-1-phenyl-1H-pyrazol-4-yl)(5-hydroxy-3-methyl-1-phenyl-1H-pyrazol-4-yl)methyl)phenoxy)hexyl)oxy)phenyl)methylene)bis(3-methyl-1-phenyl-1H-pyrazol-5-ol) **(6b)**


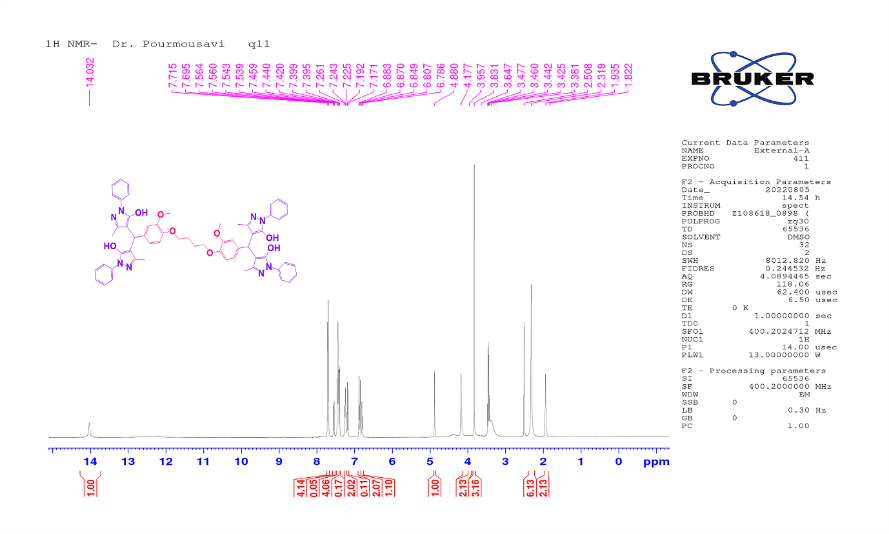


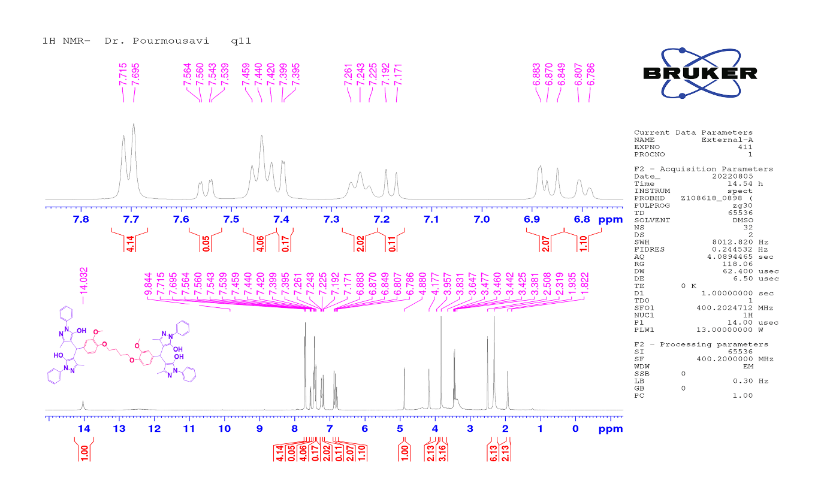


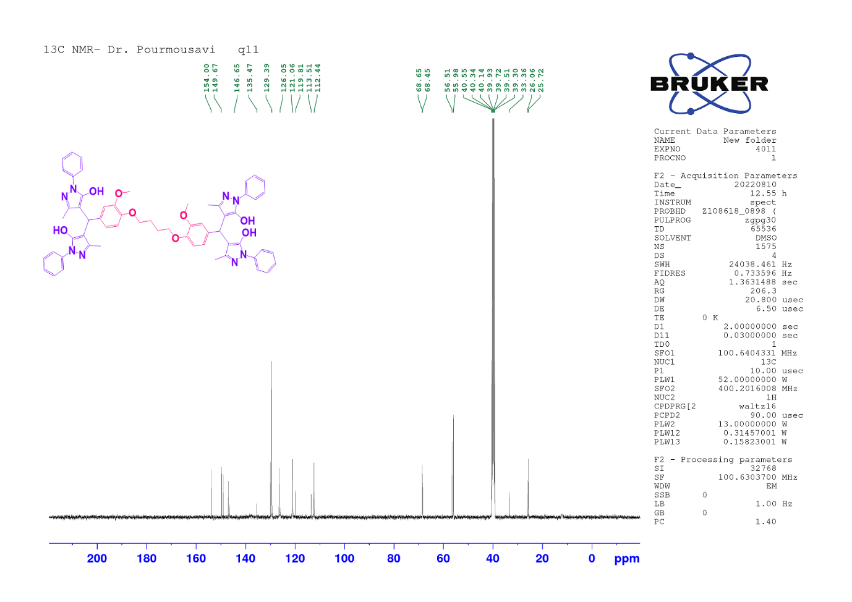


**Figure 13S.** 4,4',4'',4'''-(((butane-1,4-diylbis(oxy))bis(3-methoxy-4,1-phenylene)) bis(methanetriyl)) tetrakis(3-methyl-1-phenyl-1H-pyrazol-5-ol)**(6c)**


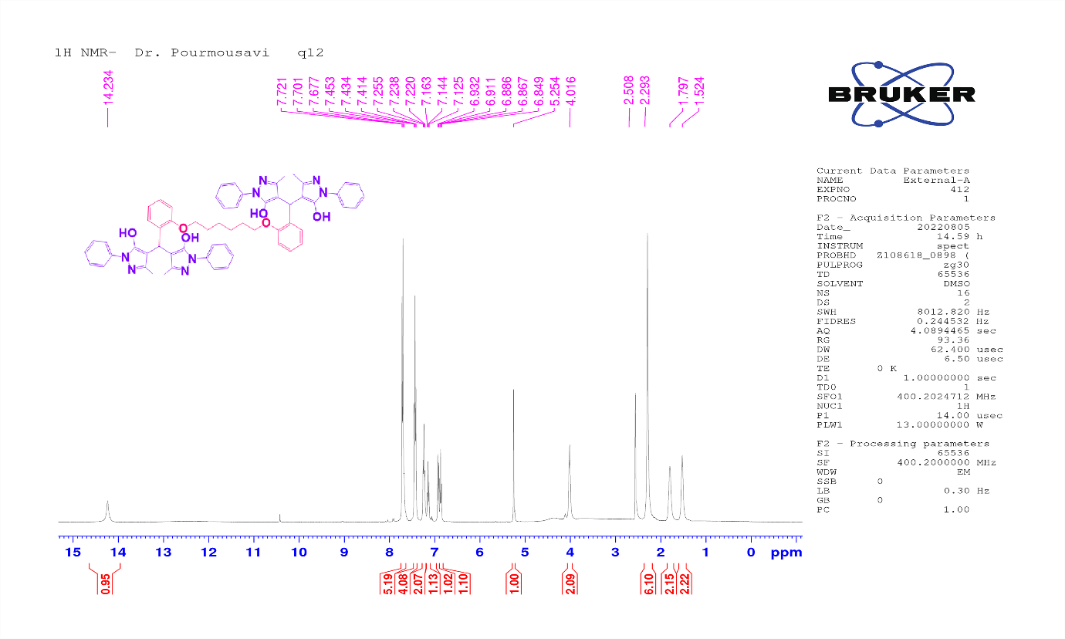


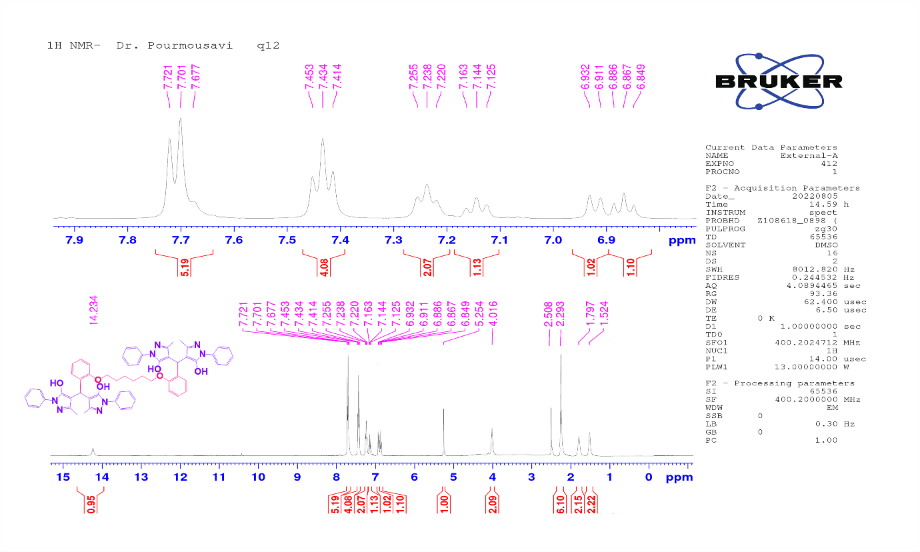


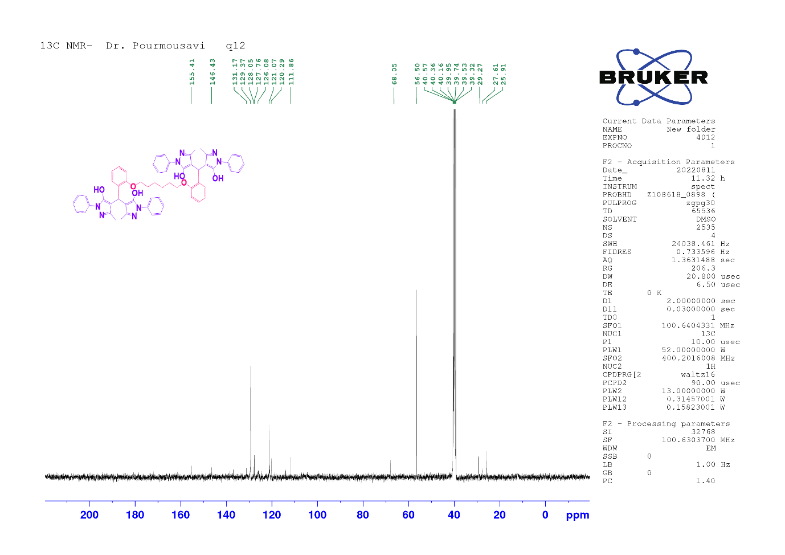


**Figure 14S.** 4,4',4'',4'''-(((hexane-1,6-diylbis(oxy))bis(2,1-phenylene))bis(methanetriyl))tetrakis(3-methyl-1-phenyl-1H-pyrazol-5-ol) **(6d)**


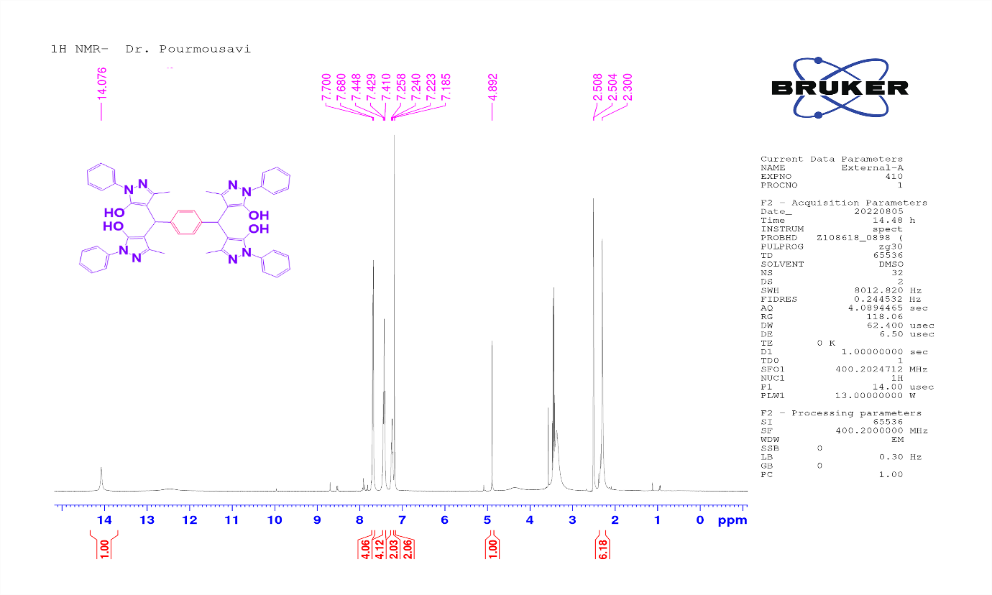


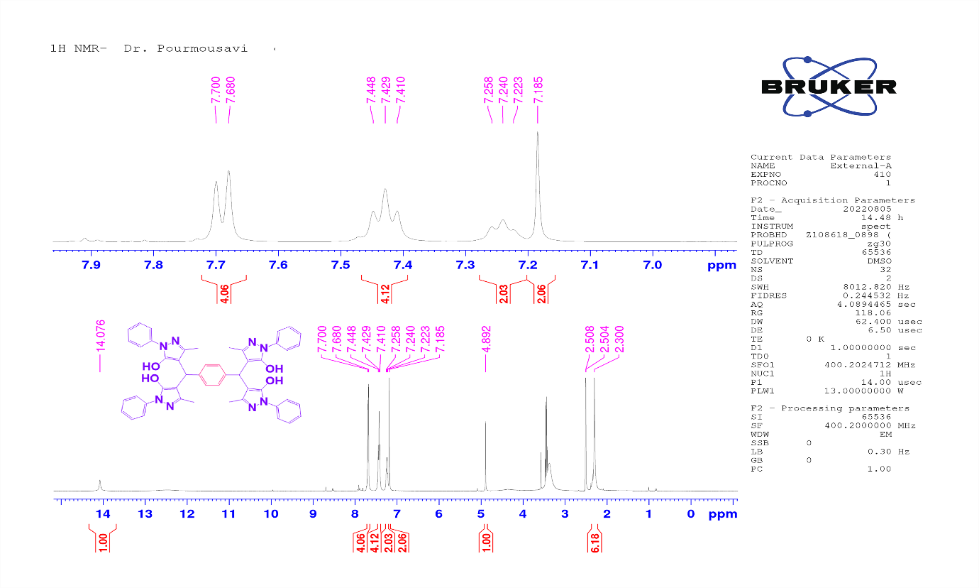


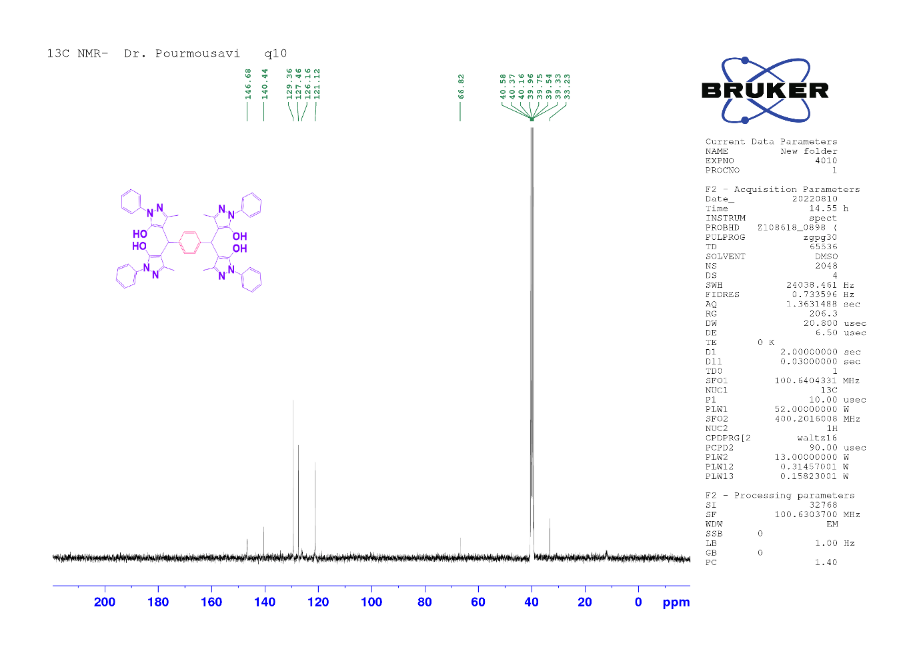


**Figure 15S.** 4,4',4'',4'''-(1,4-phenylenebis(methanetriyl))tetrakis(3-methyl-1-phenyl-1H-pyrazol-5-ol)**(6e)**

**
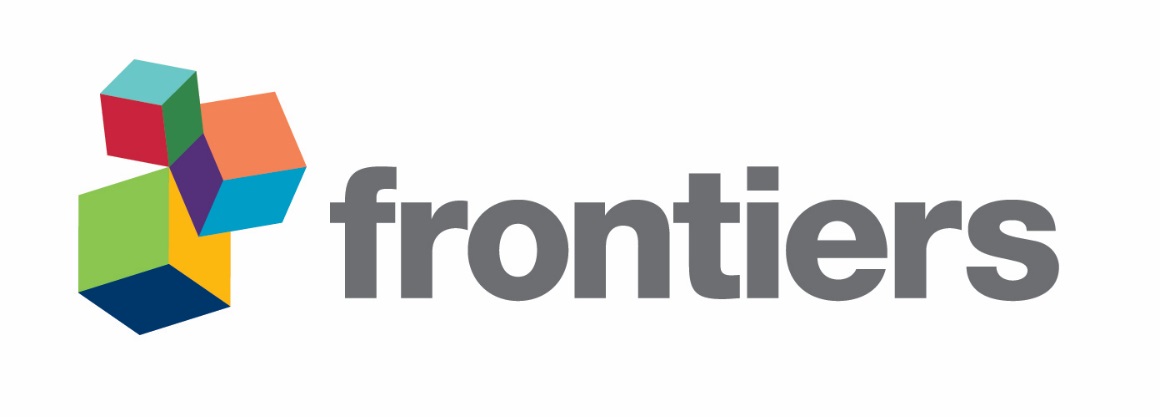
**
